# Fabrication of Flexible, Lightweight, Magnetic Mushroom Gills and Coral-Like MXene–Carbon Nanotube Nanocomposites for EMI Shielding Application

**DOI:** 10.3390/nano9040519

**Published:** 2019-04-02

**Authors:** Kanthasamy Raagulan, Ramanaskanda Braveenth, Lee Ro Lee, Joonsik Lee, Bo Mi Kim, Jai Jung Moon, Sang Bok Lee, Kyu Yun Chai

**Affiliations:** 1 Division of Bio-Nanochemistry, College of Natural Sciences, Wonkwang University, Iksan City 570-749, Korea; raagulan@live.com (K.R.); braveenth.czbt@gmail.com (R.B.); fjrtufl225@naver.com (L.R.L.); 2 Composite Research Division, Korea Institute of Materials Science, Changwon 51508, Korea; astro1228@kims.re.kr; 3 Department of Chemical Engineering, Wonkwang University, Iksan 570-749, Korea; 123456@wku.ac.kr; 4 Clean & Science Co., Ltd., Jeongeup 3 Industrial Complex 15BL, 67, 3sandan 3-gil, Buk-myeon 56136, Jeongeup-si 580-810, Korea; jjmoon@cands.kr

**Keywords:** MXene, oxidized carbon nanotube (CNTO), nanoparticle decoration, functionalization, electromagnetic interference (EMI) shielding

## Abstract

MXenes, carbon nanotubes, and nanoparticles are attractive candidates for electromagnetic interference (EMI) shielding. The composites were prepared through a filtration technique and spray coating process. The functionalization of non-woven carbon fabric is an attractive strategy. The prepared composite was characterized using X-ray photoelectron spectroscopy (XPS), X-ray diffraction (XRD), scanning electron microscope (SEM), energy-dispersive X-ray spectroscopy (EDX), and Raman spectroscopy. The MXene-oxidized carbon nanotube-sodium dodecyl sulfate composite (MXCS) exhibited 50.5 dB (99.999%), and the whole nanoparticle-based composite blocked 99.99% of the electromagnetic radiation. The functionalization increased the shielding by 15.4%. The composite possessed good thermal stability, and the maximum electric conductivity achieved was 12.5 S·cm^−1^. Thus, the composite shows excellent potential applications towards the areas such as aeronautics, mobile phones, radars, and military.

## 1. Introduction

Electromagnetic interference (EMI) leads to inevitable interactions in electronic devices. Smaller and high-speed electronic systems are susceptible to issues related to EMI which can affect either adjacent electronic items or humans, thus potentially affecting the security of the nation [1,2]. In war, electromagnetic pulse weapons are being used to affect systems utilizing electromagnetic radiation (EMR) such as radar systems, high tech complex electronic devices, remote control armor, aircraft, and missiles. In addition, EMI affects the functions of sensors in modern electronic vehicles as they transmit signals using weak radiation and microcomputers. Hence, protecting electronic devices from malfunction and achieving electromagnetic compatibility is an essential requirement around the globe. Further, electromagnetic compatibility should be attained by diminishing incoming and outgoing electromagnetic radiation, ideally without affecting the function of the devices. This is because EMI not only affects electronic systems but also causes health issues in human beings [3,4,5].

Various substances have been used for EMI shielding, such as MXenes, graphene (GN), graphene oxide, carbon nanotubes (CNTs), nanoparticles, polymers, fabrics, textiles, composites, and metals in various frequency ranges [2,4,5,6]. The EMI shielding materials can be categorized in two types: Reflection and absorption domain materials. The reflection domain materials possess mobile charges while absorption domain materials contain magnetic and dielectric materials. The layered and implanted type structures influence the EMI shielding. EMI shielding can be accomplished by suppressing the incident wave, which has the three key mechanisms of absorption, reflection, and multiple reflection. The electric conductivity associated with primary shielding factor is reflection, where the mobile electron interacts with incident wave. The thickness, electric and magnetic dipole loss, magnetic permeability, defects, and structural features induce absorption. The ohmic loss can be achieved by conduction, electron hopping, and tunneling. The polarization loss occurs due to the rearrangement of the polarization while electromagnetic radiation (EMR) is passing through the shielding materials. Polarization can be induced by embedding functionalities, hybrid fillers, nanofillers, and defects in the matrix of the composite. The inhomogeneous scattering centers, layered structure, hollow structure, and interfaces generate multiple reflection, which finally leads to absorption. Further, the skin depth limits the EMI shielding effectiveness, which should be lower than that of the thickness of EMI shielding materials [2,6,7,8,9,10,11]. Certain properties, such as being lightweight, conductive, corrosion resistant, flexible, cost-effective, and high strength, are preferable for a modern EMI shielding material. Metals are conventionally exploited as shielding materials due to the fact that they possess excellent conductivity, but they are unable to fulfill the current needs of compact electronic systems. Hence, carbon-based substances are attracting attention, as their properties can be tuned by incorporating other materials like nanoparticles and polymers [1,2,3,4,5,8]. In addition, incorporating carbon nanotubes (CNTs), MXenes, polymers, and graphene with non-woven carbon fabric significantly increases EMI shielding. Thus, establishing a conductive network is an essential factor for good EMI shielding [5,8,12]. Different combinations of the constituents and amounts of filler loading have been used to fabricate multi-functional EMI shielding materials [13,14,15,16,17].

MXenes (M_(n+1)_X_n_T_x_) are two dimensional (2D) material derived from a corresponding three dimensional (3D) MAX phase (M_(n+1)_AX_n_), where M is early transition elements (Ti, V, Cr, Nb, Ta, Zr, and Mo), A includes group 13/14 elements, X represents carbon or nitrogen, and T_x_ is surface functional groups (–OH, –F, and =O) [2]. A selective etching strategy is used in the production of MXenes. The minimally intensive layer delamination (MILD) method (LiF/HCl) has recently been endorsed, as it abridges the synthetic process, and HF, NH_4_HF_2_, and FeF_3_/HCl have also been practiced. During the etching, the weaker M–A bonds are eradicated while the strong M–C bond remains with newly formed functionalities [2,18]. MXenes have a metal-like nature, and similar to graphene, have been used for various purposes such as in sensors, capacitors, storage, and EMI shielding materials. In general, MXenes are hydrophilic in nature, as –OH is one of the surface functional groups. Thus, MXenes can be incorporated with various materials like polymers in order to tune their properties [2]. There are various types of MXenes that have been studied such as Nb_2_CT_x_, Ti_3_CNT_x_, and Ti_2_CT_x_. In addition, MXenes are used to make hybrid composites such as TiO_2_–Ti_3_C_2_T_X_/graphene, Ti_3_C_2_T_X_–sodium alginate, Ti_3_C_2_Tx/PVA, cellulose nanofibers–Ti_3_C_2_T_x_ and Ti_3_C_2_T_X_/paraffin. MXenes exhibit maximum EMI shielding of 92 dB with 45 μm thickness. Thus, the loading amount of MXenes in different polymers matrices, the morphology of composite, and the thickness influence the EMI shielding of the MXene [19,20,21,22,23].

In this study, we developed a layered pliable composite with different surface morphology and magnetic composite. Each layer of the composite consists of a layered and magnetic domain for which we employed spray coating and filtration technique under gravity. The functionalization of carbon fabric intercalation of the MXene, CNTs, and magnetic nanoparticle dramatically changed the EMI shielding. The spray coating samples were denoted as MXCNTCx, where x (x = 10, 25, or 30) was the number of coating cycles, and Ni coated fabric was expressed as MXCNTNi25. The composite fabricated using the filtration method was denoted as MXCBCM, where M is the type of metal nanoparticle such as Ni, Co, Fe, Cu, and Fe_3_O_4_. Its corresponding composites were MXCBCNi, MXCBCCo, MXCBCFe, MXCBCCu, and MXCBCFeO, respectively. Further, MXCB and MXCS were labelled based on the surfactant used, like cetyltrimethylammonium bromide (CTAB) and sodium dodecyl sulfate (SDS), respectively. MC and FC indicated uncoated carbon fabric and functionalized carbon fabric, respectively. The parameters of EMI shielding, elementals analysis, morphology, structural analysis, electric conductivity, surface property, magnetic property, and thermal stability were investigated in detail.

## 2. Materials and Methods

### 2.1. Materials

Multiwall carbon nanotube (MWCNTs) (CM-90, 90 wt %, diameter of 20 nm, and length of 100 μm) were purchased from Applied Carbon Technology Co. Ltd. (Pohang, Korea). Carbon fiber (fiber diameter 7-micron, 6 mm length) and polyethylene terephthalate (PET) binder (fiber diameter 2.2 dtex, 5 mm) were collected from TORAY product, (Tokyo, Japan). Sodium dodecyl sulfate (SDS) (98%), lithium fluoride (LiF) (98%, 300 mesh), sodium borohydride (NaBH_4_), polyacrylamide (PAM), anhydrous FeCl_2_, FeCl_3_, NiCl_2_, CuCl_2_, CoCl_2_, and cetyltrimethylammonium bromide (CTAB) were obtained from Sigma Aldrich (Seoul, Korea). Wet laid nickel coated non-woven carbon fabric (basic density of 19.2 g/m^2^, thickness of 150 µm) was acquired from Clean & Science Co. Ltd., (Seoul, Korea). Nitric acid (HNO_3_-70%) and hydrochloric acid (HCl-35%) were obtained from Samchun Chemical Co., Ltd. (Seoul, Korea). Chitooligosaccharide (Mw 5000) was issued by biomedical polymer lab, Sunchon National University (Suncheon, Korea).

### 2.2. Synthesis of Ti_3_AlC_2_

We reported that TiC, Ti, and Al powders were taken with molar ration of 2:1:1 ball milled by using Pulverisette 6 Planetary Mono Mill (Fritsch, Germany) in ethanol medium at 200 rpm for 1 h in a nitrogen environment. The resultant mixture was dried at 80 °C for 12 h. Then, 3 g of 12 mm diameter disc was prepared by applying 27.6 MPa pressure for 5 min in a laboratory press. The resultant disc was treated at 1350 °C with a heating rate of 20 °C/minute in argon gas for 2 h, then cooled down to room temperature. The treated disc was again ball milled in ethanol medium at 300 rpm for 3 h in a nitrogen environment. The powder yield was then dried at 80 °C for 12 h, and the obtained product was directly used for MXene synthesis [24].

### 2.3. Preparation of Oxidized Carbon Nanotube (CNTO)

1 g of MWCNT (CNT) and 90 mL of HNO_3_ were sonicated by using a mini ultrasonic cleaner (Uil ultrasonic Co., Ltd., Gyeonggi-do, Republic of Korea) for 5 h at room temperature. The volume of the reaction mixture was doubled by adding deionized (DI) water and filtered. The acid solution and reacted CNT were separated by filtration. The resultant black color product known as CNTO was washed until reaching a neutral pH, then dried at 80 °C for 24 h. This CNTO was used for the coating process and synthesis decorated CNTO.

### 2.4. Preparation of Fe_3_O_4_ Decorated CNTO

100 mL of 0.1 M of Fe^3+^, 100 ml of 0.1 M of Fe^2+^, and 0.5 g·L^−1^ of CNTO were stirred together at 30 °C for 30 min. A total of 10% of NH_4_OH was added dropwise until the pH of the solution reached 11–12, along with 2 mL of 0.2 M SDS. Then, the temperature of the reaction mixture was raised up to 80 °C and stirred for 2 h. The volume of the solution was reduced by half via evaporation and then cooled down to room temperature. The resultant product was washed until reaching a neutral pH in a vacuum filter, then dried at 105 °C for 12 h.

### 2.5. Preparation of Nanoparticle Decorated CNTO

100 mL of 0.1 M^2+^ of metal ion solution (Fe^2+^, Ni^2+^, Cu^2+^, and Co^2+^) and 0.5 g·L^−1^ of CNTO were stirred for 30 min. 100 mL of 0.2 M cold NaBH_4_ was added drop wisely in to the mixture, along with 0.2 g·L^−1^ of SDS. Fe, Ni, Co, and Cu decorated CNTO were successfully synthesized in a nitrogen environment. The product was filtered and washed by an ample amount of DI water. Then, the product was dried at 80 °C and 0.8 atm in a vacuum oven for 12 h. The obtained product was denoted as CM, where C is CNTO and M is nanoparticles. The corresponding decorated CNTOs were denoted as CFe, CNi, CCo, and CCu.

### 2.6. Preparation of Dispersed Solutions

0.1 g of CNTO and 0.1 g of SDS were mixed in 100 mL of deionized water and sonicated for 3 h. Then, the dispersed mixture was refluxed at 120 °C for 12 h. The obtained well dispersed solution was then used for the spray coating process. In addition, 0.4 g of CNTO and 0.4 g of CTAB were added together in 100 mL of deionized water and sonicated for 5 h (CTAB–CNTO). This procedure was repeated using SDS and CNTO (SDS–CNTO). The decorated nanoparticle dispersed solution was prepared accordingly; equal amounts of CTAB and CM (0.1 g) were sonicated in 25 mL of deionized water for 30 min. The obtained dispersed solutions were directly used for the filtration process.

### 2.7. Preparation of MXene and MXene Colloidal Solution

Equal amounts (1 g) of Ti_3_AlC_2_ and LiF were mixed together in 20 mL of 9 M HCl solution. This mixture was stirred at 35 °C for 24 h. The etched product was washed with DI water up to approximately pH 6 by centrifuging at 3500 rpm for 5 min, then Ti_3_C_2_T_x_ was dried in a vacuum oven for 12 h. Then, 0.1 g of MXene was sonicated in 10 mL of DI water in a nitrogen environment at about 15–17 °C for 2 h. The sonicated MXene solution was centrifuged at 3500 rpm for 30 min, and the supernatant was collected in a Teflon container stored at 5 °C for the coating process. The concentration of the MXene colloidal solution was 0.175 g·L^−1^. 100 mL of colloidal solution was filtered by using 0.45 μm of 47 mm of Nylon supported filter paper and dried at 80 °C (0.8 atm) until obtained constant weight. The concentration was calculated based on the weight differences.

### 2.8. Synthesis of Carbon Fabric by Wet Laid Method (MC)

We reported that 0.6 kg of carbon fiber, 0.15 kg of PET binder and 0.3 weight percent of dispersant (PAM) were dispersed in a sufficient amount of deionized (DI) water at 500 rpm for 10 min. The general wet laid method was used to produce the web. During the process, the drum dryer was used with a 140 °C surface temperature and 7 m·min^−^ speed. The areal density of obtained fabric was 30 g·m^−2^ [24].

### 2.9. Fabrication of Fabric Composite

#### 2.9.1. Preparation of Functionalized Carbon Fabric (FC)

0.2 g of chitooligosaccharide was dissolved in 100 mL of deionized water and stirred for 15 min. Then, carbon fabric with a dimension of 29 × 21 cm^2^ and a basic density of 30 g·m^−2^ was dipped in the chitooligosaccharide solution for 2 min and dried at 100 °C for 12 h. This fabric was directly used for the preparation of the filtration-based composite.

#### 2.9.2. Fabrication of MXCNTCx Composite by Spray Coating

A series of MXene–CNTO composites were prepared by the spray coating process, for which 30 g·m^−2^ of MC with a dimension of 29 × 21 cm^2^ was used, where one CNTO layer was sandwiched between two MXene layers. The thickness of the fabric was adjusted by a spraying and drying process. The drying was done using an air-drying gun while spraying was done by using an air compressor (Keyang compressors (KAC-25), Sichuan, China). This process was repeated for Ni coated fabric in order to compare the EMI shielding of carbon fabric. The coating on carbon fabric was denoted as MXCNTCx, while the Ni coated carbon fabric was denoted as MXCNTNiCx, where x was the number of coating cycles. MXCNTC30, MXCNTC25, MXCNTC10 and MXCNTNiC25 were successfully manufactured.

#### 2.9.3. Fabrication of MXene–CNTO Composite by Filtration

100 mL of MXene colloidal solution, 100 mL of CM dispersed solution and 70 mL of dispersed CNTO were alternatively filtered through FC under gravity and dried using an air gun. The resultant composite was denoted as MXCBCM. Further, MXCB was prepared by using 100 mL of MXene colloidal solution and 70 mL of CTAB–CNTO dispersed while 100 mL of MXene colloidal solution and 70 mL of SDS–CNTO dispersed mixture were used to prepare MXCS. Finally, MXCBCFeO, MXCBCFe, MXCBCNi, MXCBCCo, and MXCBCCu were successfully prepared.

### 2.10. Characterization

The structural features of the composites were investigated using a high-resolution Raman spectrophotometer (Jobin Yvon, LabRam HR Evolution (Horiba, Tokyo, Japan). A Laser Flash Apparatus LFA457 (NETZSCH, Wittelsbacherstrabe, Germany) was used to measure the density of the composites. A field emission scanning electron microscope (SEM, S-4800 (Hitachi, Tokyo, Japan) was used to examine the surface morphology of the composites. XPS with a 30–400 µm spot size at 100 W of Emax (Al anode) (K-Alpha, Thermo Fisher, East Grinstead, UK) was used to analyze the chemical environment and elemental percentage of the composites. A High-power X-ray Diffractometer D/max-2500V/PC, (Ragaku, Tokyo, Japan) with Cu(Kα) was used to record the X-ray diffraction patterns of the composites. The EMI shielding effectiveness (SE) of the composite in S-band (1–3 GHz) was recorded using an EMI shielding tent ASTM-D4935-10, ASTM International (West Kentucky, PA, USA) at room temperature while X-band (8.2–12.4 GHz) EMI shielding was measured using a vector network analyzer (VNA, Agilent N5230A, Agilent Technologies, Santa Clara, CA, USA) with a sample size of 22.16 mm × 10.16 mm. The four-probe method FPP-RS8, DASOL ENG (Seoul, Korea) was used to measure the electric conductivity of the composites. The thermal stability of the composites was tested using a Thermal Analyzer DSC TMA Q400 (TA Instruments Ltd., New Castle, DE, USA). A Mitutoyo thickness 2046S dial gage (Mitutoyo, Kanagawa, Japan) was used to measure the thickness of the composites. The surface property was measured using a Phoenix-300A contact angle meter (S.E.O.Co., Ltd., Suwon, Korea). The magnetic property was measured using SQUID—VSM (Quantum Design, Inc., San Diego, CA, USA). The graphs were plotted using a Savitzky–Golay function (Origin 2017 graphing and analysis, Origin Lab (Boston, MA, USA).

## 3. Results and Discussion

### 3.1. Structural Analysis

#### 3.1.1. Scanning Electron Microscopic Analysis of Morphology

**Figure 1:**
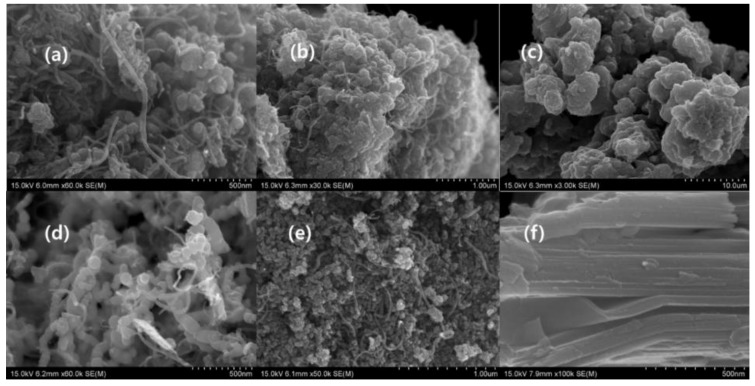
SEM image of oxidized carbon nanotubes (CNTOs) decorated by (**a**) Fe_3_O_4_ (×60,000) (**b**) Fe (×30,000) (**c**) Ni (×3000) (**d**) Co (×60,000) (**e**) Cu (×50,000) and (**f**) Ti_3_C_2_T_x_ (×100,000).

**Figure 2:**
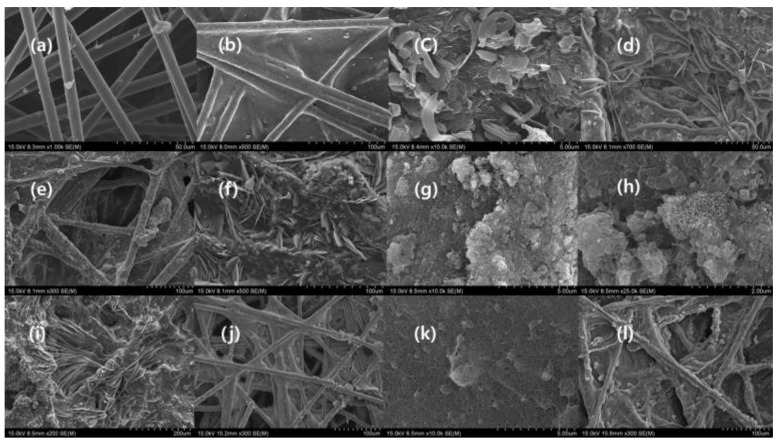
SEM image of carbon fabric composite of (**a**) Functionalized carbon fabric (FC) (×1000) (**b**) MXCB (×500) (**c**) surface of MXCS (×10,000) (**d**) MXCBFeO (×700) (**e**) MXCBFe (×500) (**f**) MXCBNi (×500) (**g**) MXCBCCo (×10,000) (**h**) MXCBCCo (×25,000) (**i**) MXCBCCu (×200) (**j**) MXCNTC25 (×300) (**k**) MXCNTC25 (×10,000) and (**l**) MXCNTNi25 (×300).

SEM was used to characterize the morphology of nanoparticles and composites, the arrangement of CNTO, nanoparticles, and MXene flakes, the structural feature of fiber, and the topography of the composites. Figure 1 illustrates the differently decorated CNTOs by different types of nanoparticles. The oxidation of the carbon nanotube mainly occurred in the tip of the carbon nanotube, as confirmed by the SEM images (Figure 1 and Figure S1), and the decoration of carbon nanotube generated a cauliflower-like structure (Figure 1a–c,e and Figure S1a,b,d). The oxidized carbon nanotube consists of a carboxylic acid functional group which can act as anchoring side of the nanoparticles. According to the Figure 1 and Figure S1, the deposition of the nanoparticle grafting occurred in the terminal of the CNTO, indicating that the oxidation predominantly happed in the tip. The oxidation of multiwall carbon nanotube produced the terminal carboxylic group which helps terminal grafting, inhibits the aggregation and increases the solubility in water. This phenomenon leads the various morphologies in decorated CNTOs [25,26]. Fe_3_O_4_, Fe, and Cu nanoparticles behaved in a similar manner, whereas the Ni nanoparticle encircled all of the carbon nanotube and was densely packed like a cauliflower. The self-assembling of the Co nanoparticles was completely different from that of another nanoparticle used. This is because it consumed CNTO as a template and formed a structure-like bacterial chain (Figure 1d and Figure S1c) [27]. The precursor of MXene, which is Ti_3_AlC_2,_ exhibited a layered structure (Figure S3h) [6]. The MILD etching created cleaves, due to the eradication of the Al layer and the evolution of the hydrogen gas (Figure 1f and Figure S3i,j). This gap is more prominent in the clay etching method (50% of HF) [18]. The etched MXene showed a layered structure with fewer gaps, and the folding of the single flake confirmed that the etching occurred. As it consisted of small gaps, it appeared like a MAX phase (Figure 1f and Figure S3i,j). Further, the presence of a single MXene flake in the composites affirmed the occurrence of effective exfoliation during the process (Figure 1f and Figure 2b–i) [27].

Figure 2 illustrates the morphology of the composites. The functionalized nonwoven carbon fabric exhibited a similar morphology of nonwoven carbon fabric, where the fibers were arranged capriciously (Figure 2b,j and Figure S3d). The fibers of the carbon fabric possessed annular gaps and cracks which were occupied by CNTO, MXene, and nanoparticle [5]. This functionalization and the nanomaterials altered the property of MC and dramatically changed the structural feature of the carbon fabric (Figure 2a–l). Filtration was an effective strategy over spray coating, because filtration closed most of the gaps between fibers and interconnected the fibers with fewer defects, while MXCNTNiC25 had prominent defects (Figure 2l and Figure S3b–j) [6]. MXCB formed like a film with well interconnected fibers, whereas few pore structures remained (Figure 2b and Figure S2b). MXene, CNTO, and Fe_3_O_4_ decorated CNTOs formed a structure like roots of a tree fixed on the soil surface, and some points of the MXene flake formed an unexfoliated MXene structure. Further, a root-like nature was given by the MXene flakes (Figure 2d). MXCBFe and MXCNTC25 formed similar structures and MXCBFe generated a highly interconnected network. In addition, the MXene flakes self-assembled in a random manner and showed a similar pattern of graphene–Polyvinylidene fluoride (PVDF) coated fabric (Figure 2c,f) [6]. Furthermore, MXCBCCu exhibited a mushroom gills-like structure which was generated by MXene flakes arranged in parallel among nanoparticles and CNTO [28]. The MXCBCCo and CNi exhibited a coral like morphology (Figure 2g,h) [29]. The coral structure was formed by cubic Co nanoparticles. In order to achieve the coral structure, CCo was used as mediator (Figure S2h,i). The surface of the MXCNTC25 displayed a network of CNTO encircling the MXene flake. Furthermore, the fibers in MXCNTNiC25 were interconnected with many defects and cracks which dramatically affected the EMI shielding and conductivity (Figure 2k,l). The etching caused the introduction of new elements such as F, Cl, and O while it eradicated most of the Al from the MAX phase, leaving Ti and C (Table S1). In addition, Cl, F, and O were derived from etchant. According to the EDX analysis, the O and C were major elements present in all composites, while other metals like Co, Ni, Fe, and Cu were also present based on the precursor used to manufacture composites. As the MXene was a structural unit of the composites, the Ti and F prevailed in most of the composites, and Al and Br were also found in some composites, which are derived from MXene colloidal solution and CTAB surfactant, respectively. In addition, exfoliated MXene had more than a single layer, which consisted of the little amount of remaining Al. Further, spray coated fabric consisted of S, which was derived from the SDS surfactant used (Table S1 and Figure S2c).

#### 3.1.2. Raman Spectroscopic Analysis for Structure of Carbon-Based Material

**Figure 3:**
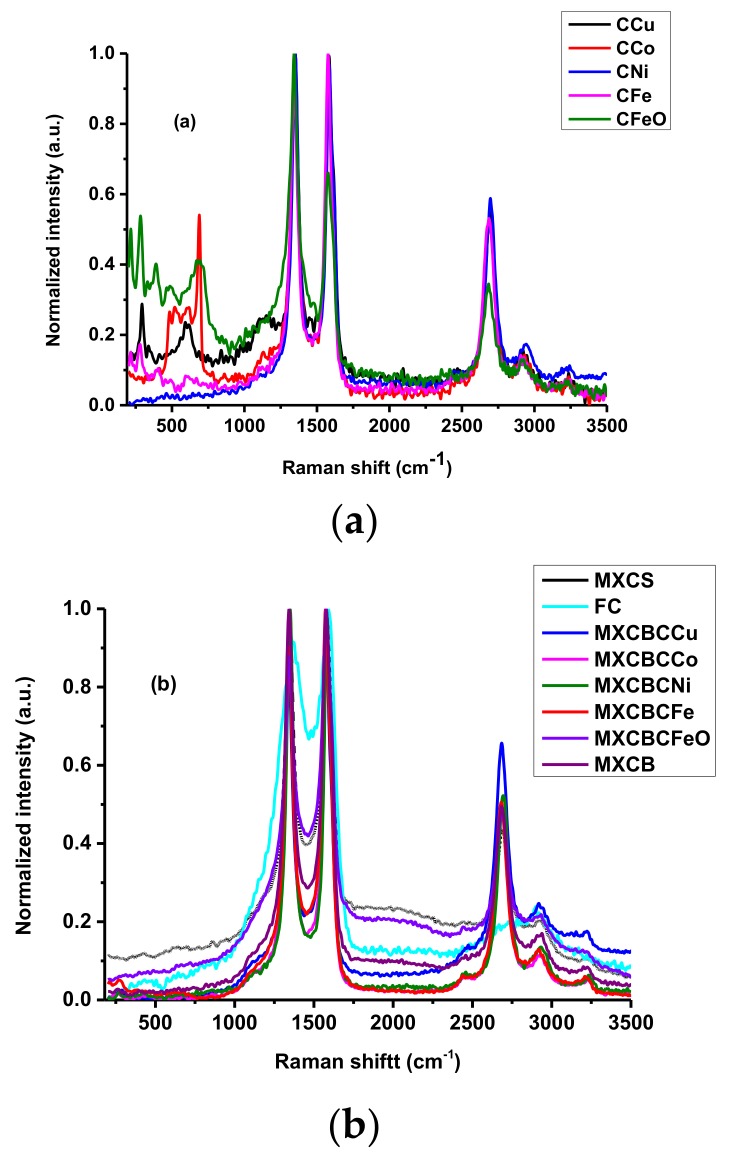
Raman spectra of (**a**) decorated carbon nanotube and (**b**) composites.

The structural and crystalline nature of materials like MXene, graphene, and CNT can be investigated using Raman spectroscopy [2,5]. Figure 3a,b illustrate the Raman spectrum of the decorated carbon nanotube and composites as plotted between 250 and 3500 cm^−1^ Raman shifts. The peaks at 624, 394, and 263 cm^−1^ were attributed to the in-plane vibrational mode of surface functionalities, C, and Ti, respectively. In addition, Ti_3_C_2_T_x_ engendered feeble wide D and G bands at 1353 and 1568 cm^−1^, respectively, and the peaks at 624, 510, and 398 cm^−1^ exhibited the presence of TiO_2_ anatase (Figure S4k) [30,31,32,33]. In addition, the missing peak at 263 cm^−1^ revealed the absence of the Al layer and the fixing of new surface functionalities in the eradicated Ti–C–Al bond. The G band and G’ band of the CNT and CNTO were located at 1570.9 and 2675.9 cm^−1^, respectively. The position of the D band slightly differed from that of the CNT peak located at 1336.3 cm^−1^ while CNTO generated a peak at 1341.5 cm^−1^. In addition, CNTO gave rise to new weak peaks at 2435.5, 2916.2, and 3320.4 cm^−1^ while CNT formed weak peaks at 2420, 2371, and 3226.2 cm^−1^. These differences were created due to the oxidation that was considered as oxidational effect of the carbon nanotube. Furthermore, the presence of defects and the amorphous nature of CNT generated the D band while the graphite structure induced the G band. The characteristic G’ band at 2672 cm^−1^ was formed by an overtone of the D band. Further, the level of defect present in the carbon nanotube can be explained using the ratio between I_D_/I_G_ and I_D_/I_G’_ [34,35,36,37,38,39,40,41,42,43,44,45]. I_D_/I_G_ and I_D_/I_G’_ of CNT were 0.79 and 1.39, respectively, while those of CNTO were 0.96 and 1.76, respectively. The CNTO possessing higher values of I_D_/I_G_ and I_D_/I_G’_ revealed that CNTO had more defect density than CNT. Hence, chemical oxidation created disorder in the carbon nanotube. The D band of the CNTO and nanoparticle decorated CNTO was located between 1340–1355 cm^−1^ while the G and G’ bands were placed between 1570–1585 cm^−1^ and 2675–2700 cm^−1^, respectively. All of the carbon nanotubes and decorated CNTOs exhibited weak peaks between 2910–2943 cm^−1^ and 3220–3240 cm^−1^, respectively. Further, there were extra peaks at lower Raman shift, which were due to the carbon–metal and oxygen–metal vibration modes (Figure 3a and Table S2) [46,47]. The G band intensity of CNTO was lower than that of CNT when compared with its corresponding D band. A similar pattern was shown by CFe whereas the other decorated CNTOs exhibited a higher D band intensity, implying that the introduction of nanoparticle generates the defect. The I_D_/I_G_ values of CCu, CCo, CNi, CFe, CFeO, and FC were 1.01, 1.15, 1.02, 0.92, 1.4, and 0.94, respectively, whereas the corresponding I_D_/I_G’_ values were 1.84, 1.96, 1.68, 1.74, 2.71, and 4.18, respectively [47]. All of the fabric showed a similar Raman spectra pattern and a peak originated between 1339–1350 cm^−1^ which was responsible for D band of the composites, whereas the corresponding G and G’ bands laid between 1567–1586 cm^−1^ and 2678–2688 cm^−1^, respectively. The non-woven carbon fabric gave rise to D and G bands at 1363 and 1592.1 cm^−1^, respectively, which is due to the graphite (HOPG), indicating the presence of the graphite-like structure and the generation of a feeble G’ band at 2908.2 cm^−1^. The I_D_/I_G_ values of the MC, MXCS, MXCBCCu, MXCBCCo, MXCBCNi, MXCBCFe, MXCBCFeO, and MXCB were 0.91, 1.03, 0.81, 0.87, 1.01, 0.86, 0.81, and 1.01, respectively, and the corresponding I_D_/I_G’_ values were 4.13, 2.33, 1.48, 2.12, 1.88, 1.96, 2.09, and 2.04, respectively (Figure 3b and Table S2). All of the composite I_D_/I_G_ values were relatively similar to MC, while the decreasing I_D_/I_G’_ value of composite confirmed that the defects of the fabric were diminished significantly. The disappearing of the lower Raman shift of MXene and decorated carbon nanotube and the formation of the new peaks confirmed that the proper link occurred between the fabric, MXene, carbon nanotube, and nanoparticles.

#### 3.1.3. X-ray Diffraction (XRD) Analysis

**Figure 4:**
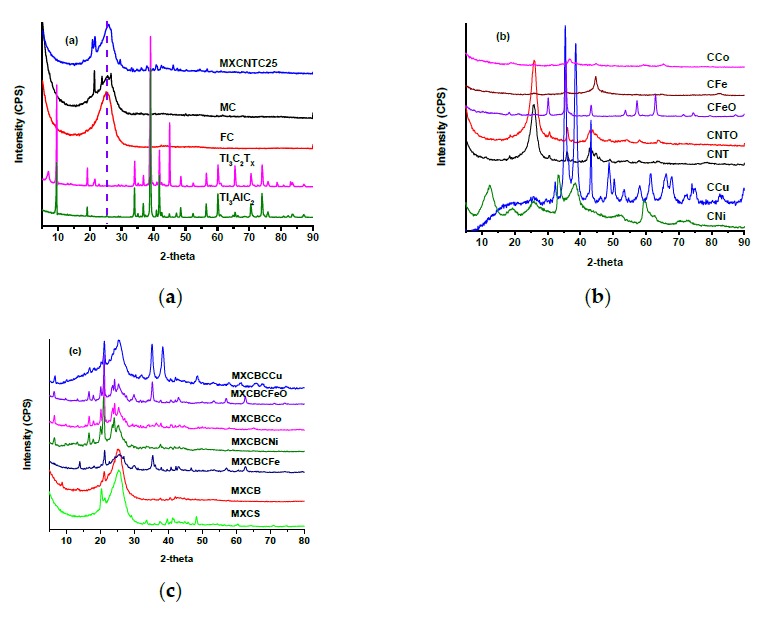
XRD of (**a**) MXene, MAX phase, and fabric (**b**) decorated carbon nanotubes and (**c**) composites.

The crystalline or amorphous nature of the material can be predicted based on XRD profile. The XRD profiles of the Ti_3_AlC_2_, Ti_3_C_2_T_x_, CNT, CNTO, decorated CNTO, and composites are shown in Figure 4a,b, and drew a 2θ range between 5 to 90°. The crystalline MAX phase generated sharp peaks at 9.52° (002), 19.53° (004), 34° (101), 35.1° (102), 36.8° (103), 38.99° (008), 41.76° (104), 42.54° (105), 48.48° (107), 52.36° (108), 56.5° (109), 60.16° (110), 52.36° (1011), 64.98° (1011), 70.34° (1012), 74.02° (118), and other miscellaneous small peaks [30,31,32,33,34,35,36]. Following the etching process, the corresponding MAX phase peaks vanished or shifted, and the sequence of new diffraction peaks was formed. The formed MXene held a crystalline nature and the peak at 7.14° (002) was a characteristic peak of MXene interplanar crystal space. In addition, the peaks originating at 14.36°, 19.12°, 28.98°, 38.86°, and 40.9° confirmed the crystalline nature of the MXene and attested to the occurrence of etching [32,33,34,35]. The peak shift of Ti_3_AlC_2_ from 9.59° to 6.96° and the formation of the MXene new peak at 21.57° indicated that the effective eradication of Al layers occurred (Figure 4a). The peak at 38.86° implied the remains of the layered MAX phase structure without an Al layer which confirmed the formation of MXene. Further, the separation of the layers after the etching was low; thus, the crystalline nature of the MXene remained the same as the structure of Ti_3_AlC_2_ (Figure 1f and Figure 4a). The carbon nanotubes exhibited a crystalline nature, as confirmed by the 25.88° (002), 42.84° (100), 43.69° (101) 48.94° (102), and 54.07° (004) reflection peaks and implied the presence of the concentric cylindrical MWCNT [48,49]. In addition, the shifting of the position of 2θ of the corresponding MWCNT attested to the oxidation of MWCNT and increased the percentage of the sp2 hybridized carbons (Figure 4b) [36,37,38,39,40]. The CFeO generated peaks at 18.36°, 30.21°, 35.66°, 43.33°, 53.76°, 57.27°, 62.88°, 71.37°, and 74.42°, and its corresponding reflection plans were (111), (220), (311), (222), (422), (511), (440), and (533), respectively [50,51]. The CFe generated peaks of zero valent iron nanoparticle at 44.73° (110), 64.53° (200), and 82.39° (211), and the other peaks were corresponding CNTO signals [52]. The (200) reflection peaks of CNi, CCo, and CCu were located at 51.68°, 51.68°, and 50.31°, respectively. The (111) peak of CCo and CCu originated at 44.87° and 43.2°, respectively [53,54,55]. The new peaks were raised due to the CNTOs and the aggregation of nanoparticles in the CNTOs (Figure 4b).

The broad peaks of MC exhibited the amorphous nature of the fabric along with the 2θ peak at 25.52°, which is similar to the peak of carbon nanotubes and confirmed the presence of a graphite structure [5,40]. Further, MC consists of various small peaks which were not above 30°, and all small peaks disappeared with functionalization (Figure 4a). The MXene–fabric composite showed various small peaks, which were absent in FC. Hence, the introduction of the MXene, CNTOs, and nanoparticles generated many small peaks. All of the carbon fabric composites showed distinctive peaks between 20.3–21.24°, and all of the nanocomposites showed characteristic peaks at 6.69° and 16.82°, except for MXCBCFe, which showed a typical signal at 14°. MXCBCCu, MXCBCFe, and MXCBCFeO exhibited a peak at 35.25° among the MXCBCCu generated shoulder peak at 38.46°. Thus, these composites can easily be distinguished from these characteristic peaks and formed from the constitutional elements of the composites.

#### 3.1.4. X-ray Photoelectron Spectroscopy Analysis

**Figure 5:**
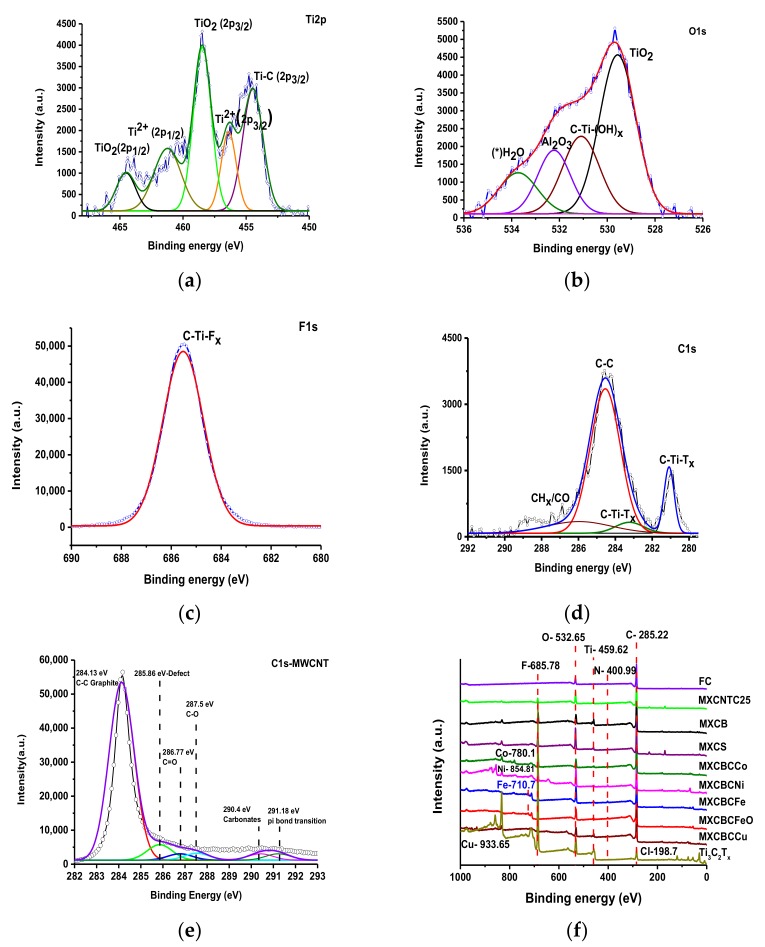
XPS fitting curves of (**a**) Ti2p, (**b**) O1s, (**c**) C1s, (**d**) F1s, (**e**) C1s of MWCNT, and (**f**) survey of the composites.

The functionalities, surface elemental composition, structure, and bonding nature of the composites can be explained using XPS. The fitting curve of MXene was plotted using overlapping curves of the Gaussian–Lorentzian function and the overlapping curve of the composites was plotted using origin pro. The fitting curves of Ti2p, O1s, and C1s, the F1s of TI_3_C_2_T_X_, and the C1s of CNTO displayed different peak positions and corresponding functional groups and bonds (Figure 5 and Table S2) [56,57,58]. The Ti2p fitting curve disclosed five different chemical environments and the corresponding binding energies were 454.5, 456.4, 458.5, 461.3, and 464.5 eV. These binding energies indicated the presence of functional groups such as Ti–C (2p_3/2_), Ti^2+^(2p_3/2_), TiO_2_ (2p_3/2_), Ti^2+^ (2p_1/2_), and TiO_2_(2p_1/2_), respectively (Figure 5a). The binding energies positions of the O1s fitting curves such as 529.6, 531.1, 532.3, and 533.8 eV showed corresponding functional groups like TiO_2_, C–Ti–Ox, Al_2_O_3_, and H_2_O, respectively, (Figure 5b–d). The presence of C–Ti–Fx generated a single peak at 685.5 eV in the F1s fitting curve (Figure 5c). The C1s fitting curves at 281.1, 283.2, 284.5, and 286.1 eV confirmed the functionalities such as C–Ti–Tx, C–C, and CHx–CO. (Figure 5d) [33,35,43,59,60,61,62]. Thus, MXene formed with the formula of Ti_3_C_2_(OH, F). In the fitting curve of MWCNT, the intense peak at 284.13 eV was raised due to the C–C bond of graphite, while the presence of the oxygen generated weaker peaks between 287–291 eV, and its corresponding oxygenic species such as C=O, C–O, and carbonate were triggered. The fitting peak at 285.86 eV confirmed the presence of the defects, which was further backed by the I_D_/I_G_ ratio and amount of oxygen [63,64]. The defects were comparatively low in CNT and increased by oxidation in CNTO (Figure 5e, Figure S4 and Table S3) [5]. The overlapping curve of XPS exhibited the constitutional elements and its corresponding peak positions. The constitutional elements of MXene were Cl, C, F, Ti, and O, and the corresponding binding energies were 198.7, 285.22, 685.78, 459.62, and 532.65 eV, respectively, while those given by N, Co, Ni, Fe, and Cu were 400.99, 780.1, 854.81, 710.7, and 933.65 eV, respectively (Figure 5f). In addition, the overlapping curve of the composite lying between 284.3–284.5 eV confirmed that the proper bonding occurred among the constitutional components (Figure S5) [56].

### 3.2. Electrical Conductivity (EC) and Surface Properties

**Figure 6:**
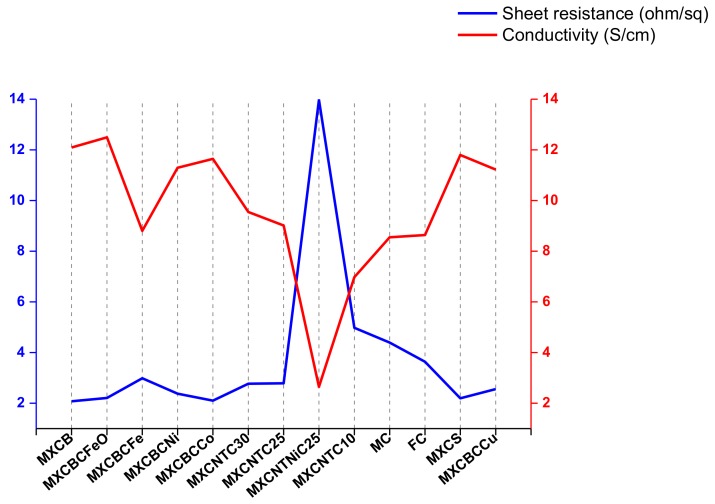
Electric conductivities and sheet resistances of the composites.

CNT and MXene possess good EC, which is one of the factors influencing the EMI shielding effectiveness [2,57]. The electric conductivity and sheet resistance (R_s_) of the fabric significantly changed due to the introduction of the MXene, CNTOs, and nanoparticles in the non-woven fabric matrix (Figure 6). The fabricated composite exhibited EC ranging from 12.5 to 2.65 S·cm^−1^, and R_s_ lay between 13.98 and 2.08 Ω·sq^−1^. The MXCNTNiC25 hit a maximum R_s_ of 13.98 Ω·sq^−1^ and a minimum conductivity of 2.65 S·cm^−1^, which was due to the high surface defect (Figure 2l). The functionalization process increased EC by 10.1% and changed R_s_ by 20.9%. Thus, the functionalization process minimized the defect and increased the electron mobility. The filtration-based composite showed EC above 10 S·cm^−1^ while MXCBCFeO and MXCBCFe exhibited a maximum and minimum of 12.5 and 8.81 S·cm^−1^, respectively. The EC of MXCB, MXCBCNi, MXCBCCo, MXCS, and MXCBCCu were 12.1, 11.3, 11.65, 11.8, and 11.22 S·cm^−1^, respectively, whereas the corresponding R_s_ were 2.08, 2.38, 2.11, 2.2, and 2.56 Ω·sq^−1^, respectively. Spray coated MXene–CNTO composites displayed EC below 10 S·cm^−1^, among which MXCNTC30 exhibited 9.55 S·cm^−1^. Hence, 14.62% of electric conductivity was increased by introducing MXene, CNTOs, and decorated carbon nanotubes into the MC network (Figure 6, Figure S6 and Table S6). The MXene has a high instinct electric conductivity due to its metal like nature [5]. In addition, the doping of CNT by nitrogen expands the EC while the degree of oxidation reduces the conductivity. However, the MXene–carbon fabric composite showed a maximum of 8.84 S·cm^−1^ EC while the CNTO-carbon fabric composite displayed 16.32 S·cm^−1^ of EC. Thus, MXene–CNTO-nanocomposite showed conductivity between the MXene and CNTO composites [2,5,24,65]. These defects limit the electron mobility and electron hopping along the fiber [66,67]. We attempted to explain this using the hydrophobic nature of the composites. The wetting ability of the surface can be explained based on the contact angle, which above 90° is called a water-repellent surface, while below 90° is considered a water loving surface. Further, the contact angle is dependent on the surface roughness and energy. Roughness increases the defect, thus increasing the surface energy and surface roughness raise the hydrophobic nature [5,16,56]. The contact angle, wetting energy, spreading coefficient, and work of adhesion of MXCNTNiC25 were 131.3°, −48.09 mN·m^−1^, −120.89 mN·m^−1^, and, 24.71 mN·m^−1^, respectively, while FC exhibited 134.18 mN·m^−1^, −50.7 mN·m^−1^, −123.54 mN·m^−1^, and 22.06 mN·m^−1^, respectively. In addition, the other composites showed no coated angle that was due to the absorption of the water or well spread on the surface of the composites. The conductivities of most of the composites were high considering the fewer surface defects while the conductivity of the MXCNTNiC25 showed the lowest conductivity, confirming that MXCNTNiC25 possessed high defect and surface roughness (Figure 2l and Figure 6, and Figure S7).

### 3.3. Magnetic Properties of the Composites

**Figure 7:**
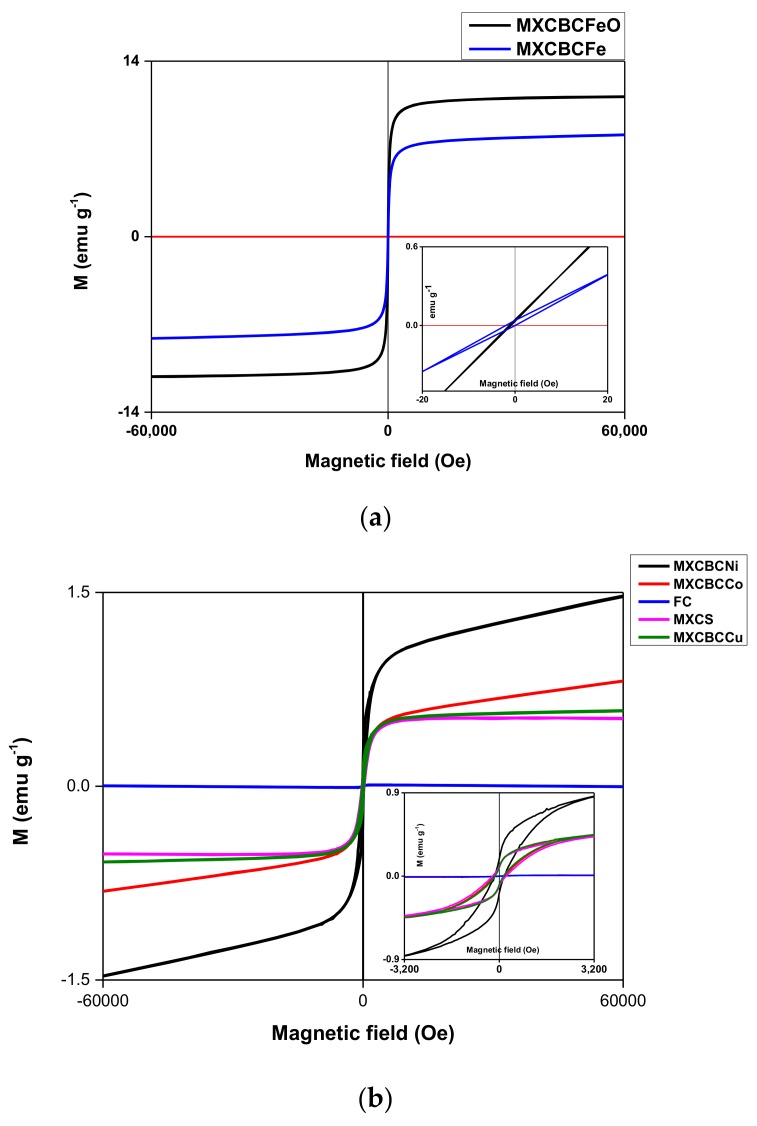
Magnetization against applied field at 300 K: (**a**) Fe- and Fe_3_O_4_-based composites and (**b**) Ni, Co, Cu, and non-nanoparticle-based composites.

The magnetic properties of the composites at 300 K were studied using hysteresis loop measured at 300 K (Figure 7 and Figure S8). The saturation magnetization (Ms), remanence (Mr), coercivity (Hc), and coefficient of squareness of hysteresis loops (Kp) can be determined using hysteresis loop [68,69,70,71]. In addition, the Kp can be calculated using the Mr/Ms ratio. Alborzi et al. and co-workers showed that decreasing the Kp value enhanced the super magnetic property, whereas diminishing the particle size decreased the Hc and increased the Ms. In addition, super magnetic material can be created by minimizing the Hc [69]. The formation of a cluster structure improves the Ms and dropping the cluster size leads to lower magnetic energy and super magnetic behavior [71]. Thus, the magnetic properties of the materials were influenced by various factors such as the geometry, size, functional groups, morphology, and crystallinity [72]. Further, precursor salt also affects the magnetic properties of the material and the synthesis of Fe_3_O_4_ by using ferrous and ferric sulfate generates 46.7 emu·g^−1^ of Ms while ferrous and ferric chloride produce 55.4 emu·g^−1^ of Ms and the magnetization of bulk Fe_3_O_4_ is 93 emu·g^−1^ [69]. All the composites possessed nonlinear behavior against applied field and showed the hysteresis loop (Figure 7 and Figure S8). The Ms of the composites ranged from 0.45–0.009 emu·g^−1^ and the saturated magnetic strength was placed between 9.95–3284.4 Oe. The Kp ranging from 0.022 to 1.128 and Mr fluctuated between 2.2 × 10^−4^–0.187 emu·g^−1^, whereas a 1.27–232.3 Oe range of Hc was given by the composites (Figure 7, Figure S8 and Table 1). According to Figure 7b, the magnetization of the FC approached almost zero, compared with other composites, and the hysteresis loop was not smooth, like other composites. It was considered to be due to the irregular arrangement of the fibers (Figure S8c and Figure 2a), and a study by Lu et al. showed that carbon fibers are non-magnetic materials. Thus, functionalization induced the magnetic behavior of the MC [7]. Furthermore, the Ms, Mr, and Kp values were lower than those of the others. This confirmed that interconnecting fibers using nanomaterials alters the magnetic property of the nonwoven fabric (Table 1 and Figure 2b–l). MXCBCNi and MXCBCCo behaved differently when applied magnetic field increased the magnetization and also increased while others exhibited constant magnetization (Figure 7b). A part of the loop of MXCBCFe pass through the origin, did not have negative Mr and positive Hc value, and had more than one loop, whereas MXCBCFeO did not have a positive Hc value, and instead the loop was located near the Mr point. Further, MXCBCFeO and MXCBCFe possessed high Kp values of 1.128 and 0.095, where 0.095 was lowest among all composites. Additionally, MXCBCFeO and MXCBCFe contained the lowest Ms, Hc, and saturated magnetic strength, which affect the EMI shielding of the nanoparticle-based composites. According to this study, increasing Kp increased the EMI shielding.

### 3.4. Electromagnetic Shielding Effectiveness (EMI-SE) of Composites

**Figure 8:**
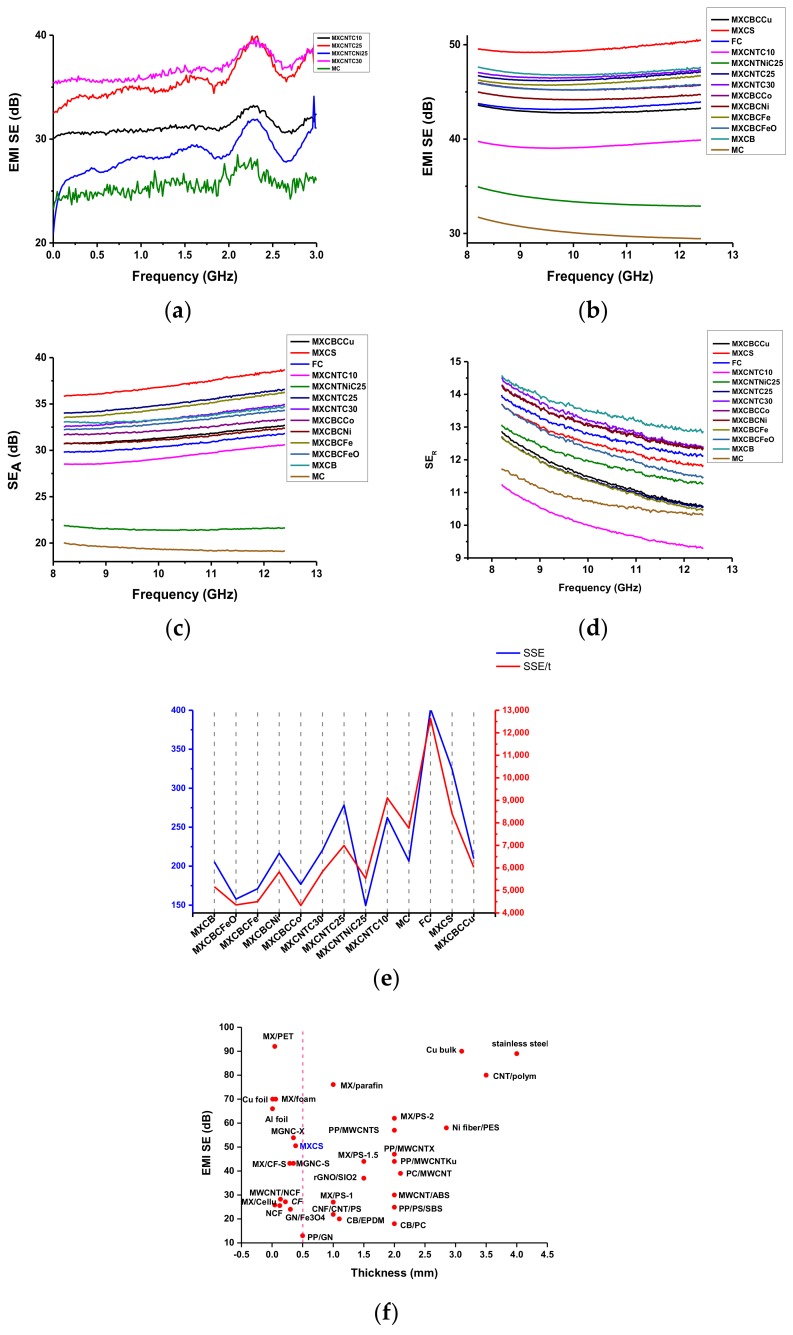
Electromagnetic interference (EMI) shielding of the composites: (**a**) Total EMI shielding (S band), (**b**) total EMI shielding (X band), (**c**) absorption (X band), (**d**) reflection (X band), (**e**) specific shielding effectiveness (SSE) and absolute effectiveness (SSE/t) of the composites, and (**f**) EMI shielding comparison of different composites.

The study employed filtration and spray coating techniques, for which 30 g·m^−2^ of areal density fabrics were used, while Ni coated fabric was 20 g·m^−2^ of areal density. For the spray coating, MXene colloidal solution and dispersed CNTO and SDS solution were used, while each coating used 40 mL of solution. A maximum of 100 mL of colloidal solution was used in the filtration process. In spray coating composites, one MXene layer was sandwiched between two layers of CNTO, while one MXene layer placed between CNTO and nanoparticles decorated CNTO in filtrated composites. For the spray coating process, SDS was used as surfactant and CTAB was used to disperse nanoparticles decorated carbon nanotube. The spraying and filtration process changed the thickness and pore size of the fabric. The EMI shielding was performed in the region of the X band and S band. X band (8–12.4 GHz) EMI-SE was performed for all composite, while S band (1–3 GHz) EMI-SE was only measured for spray coated composites (Figure 7a). The intercalation of nanomaterials with FC and MC significantly increased the EMI shielding. The spray coated composite showed a higher EMI-SE between 1.5 to 2.6 GHz and non-woven carbon fabric preferable over Ni coated carbon fabric as it showed a lower EMI-SE than the other composites (Figure 8a,b). The non-fabrics were flexible and contained physically interconnected fibers with a three-dimensional reticular structure. When the incident wave hit the surface of the shielding materials, the reflection, multiple reflection, absorption, and transmission occurred. The strength of this mechanism varies based on the materials used for EMI-SE. According to the Simon formalism, EMI-SE depends on the electric conductivity and thickness of the material, and the length of the carbon fiber has no influence on EMI shielding. Further, hiking areal and volume density increase the electric conductivity and EMI shielding. A study by Lu et al. showed that the EMI-SE of 50 g·m^−2^ of carbon fabric is 30.2 dB, while 30 g·m^−2^ produces 23.1 dB, and the EMI shielding of carbon fabric is independent of the frequency range [7,73,74]. Hence, increasing the areal density of the carbon fabric increases the EMI-SE. The 30 g·m^−2^ of MC gave rise to a maximum of 28.5 dB of EMI-SE in the S-band whereas 31.7 dB of EMI-SE was generated in the X-band region, and this was further enhanced by functionalization up to 43.9 dB of maximum (Table S5 and Figure 8a,b). This result was almost consistent with that of the Lu et al. study that EMI-SE was independent of frequency. This is despite the fact that the areal density and electric conductivity is low while functionalization had a greater effect on EMI-SE. After the functionalization, the MC possessing the magnetic property was advanced criteria for the high EMI-SE.

All the FC-based nanocomposites’ minimum, maximum, and averaged shielding were above 99.99% of incident wave in the X-band region while spray coated composites’ maximum shielding was just below 99.99% and the minimum and average laid between 99–99.9%. For the FC-based composite of SE, reflection (SE_R_) and absorption (SE_A_) prevented 90% and 99.9%, respectively. MXCNTC30 and MXCNTC25 displayed 99.99% of maximum, minimum, and average shielding whereas the others showed 99.9% of shielding in the X-band region. In addition, spray coated composites SE_A_ were in the range of 99–99.9% and 90% of reflection. The spray coated composite showed maximum shielding of 99.9% (Figure 8a–d and Table S5). The maximum EMI-SE shown by MXCNTC30, MXCNTC25, MXCNTNiC25, MXCNTC10, and MC in the S band were 39.6, 39.9, 34.1, 33.2, and 28.5 dB, respectively, whereas 47.3, 47.1, 34.9, 39.9, and 39.6 dB were given in the X-band region. Thus, the shielding of composites comparatively increased when the measurement frequency reached from the S band to the X band. Further, it was obvious that above 25 coating cycles, the shielding of composite was significantly reduced in the S-band region. It was considered that increasing the amount of MXene and CNTO reduced the conductivity and shielding ability of composite in the S band. When MXCNTC30 went from the S band to the X band, the EMI-SE increased by 19.4%, while it increased by 18% for MXCNTC25. (Figure 7a and Table S5). Thus, EMI-SE for nanocomposites were studied in the X-band region. By contrast, MXCNTNiC25 showed the lowest shielding in the S and X bands, due to surface defects and low electric conductivity. The defect was due to the reduced adhesive ability of MXene–CNTO composites with Ni coated carbon fabric, and the carbon fabric showed low defects (Figure 2l and Figure 8 and Figure S3f). The MXCNTNiC25 exhibited higher EMI shielding than MC as the porosity was diminished. Nonetheless, all the coated carbon fabric showed higher EMI shielding than MXCNTNiC25 [1,2,3]. The maximum EMI-SE of MXCB, MXCBCFeO, MXCBCFe, MXCBCNi, MXCBCCo, MXCBCCu, and MXCS were 47.6, 45.9, 46.7, 45, 46, 43.6, and 50.5 dB, respectively. The nanoparticle-free composites displayed higher blocking ability than others and the mushroom gills like structure significantly reduced the EMI-SE as compared with FC, whereas the coral like structure showed a higher EMI-SE than FC (Figure 2g–i and Figure 8b and Table S5). Despite this, all showed above 45 dB, except for MXCBCCu (Figure 7 and Table S5). The absorption was dominant over reflection, and reflection stayed nearly constant for all composites. Thus, the changing of the shielding effectiveness was due to the absorption of the composites. The composite showed electric conductivity in the range of 2–13 S·cm^−1^, which was not sufficient to produce good SE_R_. The effective percolation of the conductive network facilitates the electron mobility and reflection and enhances the ohmic loss [1,2,3,7]. The intercalation of the MXene, CNTO, and nanoparticle decorated CNTO increase the electric conductivity, though the lack of an effective conductive network minimizes the SE_R_ [7]. In this case, part of the incident wave was reflected, while most of the remaining part underwent absorption and multiple reflection. The maximum SE_A_ of MXCB, MXCBCFeO, MXCBCFe, MXCBCCo, MXCS, MXCNTC30, and MXCNTC25, were all above 33 dB, while the SE_R_ of all the composites were above 10 dB (Figure 8c,d and Table S5). In addition, the Kp value above 0.09 led to shielding above 99.99 % (Figure 7a,b and Table 1). Thus, absorption can be achieved by different internal geometry, functionalization, ohmic loss, defects, and magnetic property of materials. In addition, the CNTO contributed inner tube scattering leads to a higher SE_A_ because the blend of different components establishes diverse phases which enhance multiple reflection and absorption [1]. The functionalization considerably increased the specific shielding effectiveness (SSE) and absolute effectiveness (SSE/t), and the SSE and SSE/t of the FC were 401.93 dB cm^−3^·g^−1^ and 12639.95 dB cm^−2^·g^−1^, respectively. The intercalations of zero, one-, and two-dimensional materials in the three-dimensional fiber network significantly reduced the SSE and SSE/t. Further, iron-based composited displayed lower SSE and comparatively lower SSE/t (Figure 8e and Table S6).

Figure 8f and Table S4 compared the EMI-SE with previous corresponding work. The dip coated CNTO non-woven fabric (basic weight 20 g·m^−2^) showed a maximum of 33 dB, which is substantially lower than that of the MXene–CNTO–nanoparticle composite [5]. The polystyrene–MWCNT composite showed a maximum EMI-SE of about 22 dB. The Polyvinylidene fluoride (PVDF)–MWCNT composite exhibited 28.5 dB of EMI-SE with a thickness of 0.2 cm while the segregated carbon nanotube–polypropylene composite showed an EMI-SE of 48.3 dB with 0.22 cm of thickness and interconnected MWCNT polymeric matrix exhibited 27 dB at 18 GHz [1,12,13,74,75]. In addition, the spongy carbon nanotube composite disclosed 54.8 with thickness of 0.18 cm in the X–band region [16]. Most of the study showed the EMI shielding range of the CNT composite was 20–30 dB with higher thickness. The different types of fillers and geometry altered the shielding ability of the MWCNT. Thus, we analyzed various combinations of materials with different geometries in order to increase the shielding effectiveness of the non-woven fabric and MWCNT. MXene film crammed between Polyethylene terephthalate polymer film showed an excellent EMI shielding of 92 dB with 4600 S·cm^−1^ electrical conductivity [2]. Cao et al. and coworkers highlighted that nacre-like MXene–cellulose nanofiber composite showed an EMI shielding range of 5.3–25.8 dB based on the percentage of MXene added to one dimensional cellulose fiber [76]. The MXene–carbon fabric composite exhibited an EMI-SE of 43.2 dB [24]. Hence, a combination of the MXene–CNTO-based composite dramatically increased the EMI-SE. Further, the carbon fabric-based composite showed lower shielding; therefore, the manufactured composite was considered to have excellent EMI-SE ability (Figure 8f).

### 3.5. Thermal Stability and Thermo Gravimetric Analysis of Composites.

**Figure 9:**
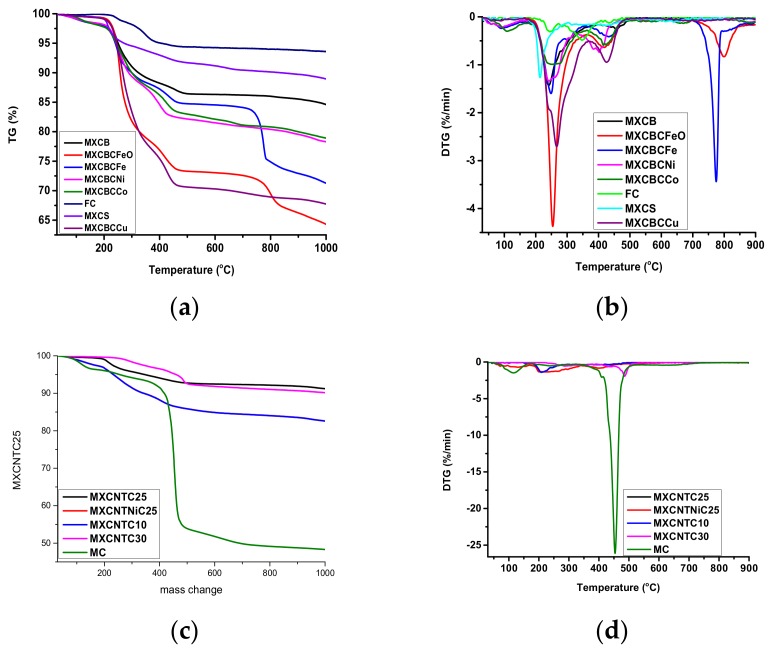
TGA and differential thermal analysis (DTA) analysis of the composites (**a**) TGA of filtration-based composite, (**b**) TGA of filtration-based composite, (**c**) TGA of spray coated composite, and (**d**) DTA of spray coated composite.

Thermogravimetric analysis (TGA) and differential thermal analysis (DTA) were used to investigate the thermal stabilities of the composites. TGA graphs were plotted in the temperature range of 30–1000 °C while DTA graphs were plotted in the range of 30–900 °C (Figure 9a–d). The TGA and DTA analysis were carried out using Al_2_O_3_ crucible in a nitrogen environment with a heating rate of 10 °C·min^−1^. The composites exhibited an outstanding thermal stability over 100 °C. Most of the composites had a starting point of degradation above 140 °C, and the degradation beginning lay between 120–255 °C, where MXCNTC30 showed 120 °C. In addition, MXCB, MXCBCFeO, MXCBCFe, MXCBCNi, MXCBCCo, and MXCNTNiC25 prohibited degradation above 150 °C among mushroom gill like MXCBCCu decomposed at lower temperature of 127.5 °C, whereas the degradation temperatures of the other composites were below 149 °C and above 127.5 °C (except MXCNTC30 at 120 °C). The rapid mass changing percentage of composites occurred between 120–897.5 °C where spray coated composite hit the higher value (above 700 °C), while filtered gave rise below 653 °C (Figure 2i and Figure 9a,c and Table S7). The temperature between the 30–1000 °C range of weight losing percentage of the composites were 6–36%, in which MXCBCFeO gave a maximum of 35.71% while 8.85% of weight loss occurred in MXCNTC25 (Figure 9a,c and Table S7).The MC and FC degradations of the whole temperature range were 6.84% and 6.4%, respectively, and the corresponding degradation beginning temperatures were 190 °C and 255.5 °C, respectively (Figure 9a,c and Table S7). The functionalization of MC by using chitiooligosaccharide increased the thermal stability. This was attributed to the fact that the chitiooligosaccharide contains lots of the hydroxyl groups, thus creating the hydrogen bonding which led to higher thermal stability [68]. According to Raagulan et al., rapid mass changes occurred due to the loss of surface functionalities, water, and decomposition of fabric [5,6,63,64]. In addition, the MXene–carbon fabric composite prohibited degradation up to 235 °C and a Gamage et al. study showed CNTO–carbon fabric composites prohibited degradation until 284 °C. The combination of MXene, CNTO, and nanoparticles considerably diminished the degradation temperature (Table S7). The DTA analysis revealed various peaks raised due the degradation of the composites (Figure 9b,d). Strong peaks were placed in the region of the rapid changing of TGA curve [77]. Further, we observed that the slope of the TGA curve depends on the intensity of the corresponding peaks in DTA analysis, and that the high intensity of the DTA peaks lead to rapid degradation. The more intense peaks were located between 209–934.2 °C temperature range (Figure 9b,d, Figure S9a–m and Table S8). It was obvious that the introduction of the nanomaterials in the fabric network shifted the peaks to lower temperatures and diminished the decomposition temperature.

## 4. Conclusions

The filtration-based nanocomposite and spray coated nanocomposite were successfully prepared. The effect of the functionalization of non-woven carbon fabric was analyzed as well. The composites were lightweight and thinner. The density of the composites ranged from 0.108 to 0.288 g·cm^−3^ while the thickness of the composites ranged between 0.266–0.408 mm. The ranges of Ms and Mr were 0.0099–0.86 emu·g^−1^ and 0.00022–0.44 emu·g^−1^, respectively. The MXCBCNi displayed a higher Ms of 0.86 emu·g^−1^ and a 0.44 emu·g^−1^ of Mr was shown by MXCBCFeO. The MXCCFeO exhibited a maximum Kp of 1.128 while FC was 0.022. The functionalized carbon fabric displayed a hydrophobic nature with a 134.18° contact angle, whereas MXCNTC25 displayed a contact angle of 131.3°. The electric conductivities of the composites varied between 2.65–12 S/cm, whereas the surface resistances were in the range of 2.08–13.98 Ω/sq. The composites showed good thermal stability and resisted complete thermal degradation above 120°. In addition, functionalization increased the thermal stability and prevented degradation up to 255.5°. The maximum EMI-SE shown by MXCS was 50.5 dB, and the EMI shielding range of the composite was 50.5–28.5 dB. The maximum SE_R_ and SE_A_ were shown by MXCB and MXCS, respectively. The ranges of SSE and SSE/t were 149.37–401.93 dB cm^3^·g^−1^ and 4330.82–12,639.35 dB cm^2^·g^−1^, respectively, where FC showed maximums of both. Hence, the manufactured fabric composite presented high EMI shielding, magnetic behavior, low density, smaller thickness, and flexibility.

## Figures and Tables

**Table 1:** Comparison of saturation magnetization, retentivity, and coercivity of the composites.

Composites	Saturated Magnetic Strength (Oe)	Saturation Magnetization (Ms) (emu·g−1)	Remanence (Mr) (emu·g−1)	Coefficient of Squareness of Hysteresis Loops (Kp) = Mr/Ms (Dimensionless)	Coercivity (Hc) (Oe)
MXCBCCu	3284.4	0.45	0.08	0.178	144.59
MXCS	3200	0.43	0.1	0.233	232.3
FC	2587.7	0.0099	2.2 × 10−4	0.022	22.34
MXCBCCo	3195.9	0.45	0.096	0.213	185.57
MXCBCNi	3184.9	0.86	0.187	0.217	152.58
MXCBCFe	21.4	0.41	0.039	0.095	2.02
MXCBCFeO	9.95	0.39	0.44	1.128	1.27

## Supplementary Materials

The following are available online at https://www.mdpi.com/2079-4991/9/4/519/s1, Figure S1: SEM image of CNTO decorated by (a) Fe3O4 (b) Fe (c) Ni (d) Cu; Figure S2: SEM image of carbon fabric composite of (a) MC (500) (b) MXCB (500) (c) surface of MXCS (10000) (d) MXCBCFeO (500) (e)MXCBCFe (500) (f)MXCBCNi (450) (g) MXCBCCo (150) (h) MXCBCCo (100000) (i) MXXBCCu (45000) (j) MXCNTC30 (100) (k) MXCNTNi25 (1000) (l) MXCNT10 (400); Figure S3: SEM image of (a) MC (×500), (b) MC (×2000), (c) cracks on fiber (×300000), (d) MXene-CNTO coated carbon fabric (×500), (e) MXene-CNTO coated carbon fabric (×300), (f) MXene-CNTO coated fabric (Ni coated fabric) (×300), (g) MXene and CNTO on the surface of the fabric (×120000), (h) Ti3AlC2 (×22000), (i) Ti3C2Tx (×50000) and (j) Ti3C2Tx (×80000); Figure S4: Normalized curve of Raman spectrum of (a) decorated CNT, (b) composites (c) CNT, (d) CNTO, (e) CCu, (f) CCo, (g) CNi, (h) CFe, (i) CFeO, (j) FC (k) comparison of MXene, MAX phase and MC, (l) MC, (m) MXCS, (n) MXCBCCu, (o) MXCBCCo, (p) MXCBCNi, (q) MXCBCFe, (r) MXCBCFeO and (s) MXCB; Figure S5: The c1s fitting curve of the MXene, functionalized fabric and composites; Figure S6: Resistivity profile of the composites; Figure S7: Contact angle of (a) MXCNTNiC25 (b) FC and (c) represent other composites; Figure S8: The magnetization of composites against the applied field at 300 K (a) MXCBCCu, (b) MXCS, (c) FC, (d) MXCBCCo, (e) MXCBCNi, (f) MXCBCFe, (g) MXCBCFe and (h) MXCBCFeO; Figure S9: Comparison of the TG and DTA of all the composites (a) MXCB, (b) MXCBCFeO, (c) MXCBCFe, (d) MXCBCNi, (e) MXCBCCo, (f) MXCS, (g) MXCBCCu, (h) FC, (i) MXCNTC25, (j) MXCNTNiC25, (k) MXCNTC10, (l) MXCNTC30 and (m) MC; Table S1: Elemental percentage of composites from EDX analysis; Table S2: Raman spectra band positions; Table S3: Atomic percentage MXene, MC, CNT, CNTO and composites from XPS analysis; Table S4: Comparison of EMI SE with thickness; Table S5: Comparison of maximum (MAX), minimum (MINI), average (AVE) shielding, SSE and SSE/t of the composite in each case; Table S6: Density of the composites; Table S7: Comparison of mass changes with different temperature range from TGA analysis; Table S8: Comparison of different peak positions range from DTA analysis.
