# Supplementary material for: Fabrication of Flexible, Lightweight, Magnetic Mushroom Gills and Coral-Like MXene–Carbon Nanotube Nanocomposites for EMI Shielding Application
Source: Nanomaterials (Basel). 2019 Apr 2;9(4):519. doi: 10.3390/nano9040519 (PMC6523891; doi:10.3390/nano9040519)
Supplement: Supplementary file 1 [file nanomaterials-09-00519-s001.pdf]

Fabrication of Flexible, Lightweight, Magnetic Mushroom Gills and Coral-Like MXene–Carbon Nanotube Nanocomposites for EMI Shielding Application

Supplementary Texts

Electrical conductivity

The electrical conductivity reciprocally changes with resistance. The resistance of a material can be calculated as [1]

$$R = \varrho\frac{L}{A} = \varrho\,\frac{L}{Wt}$$

$$A = wt$$

Where R is the material resistance, ϱ is the resistivity, A is the cross-sectional area, L is the length. W is width and t is the thickness

$$R= \frac{\rho L}{tW} = R_s\,\frac{L}{W}$$

Where,

R_s - Sheet resistance

Bulk resistivity ϱ (in Ω cm) can be obtained by the following equation.:

$$\rho = R_s{\cdot}t$$

The conductivity of the materials can be defined as. σ = 1/ ρ where, the conductivity of the material can be given as,

$$\sigma = \frac{1}{\rho}$$

$$\sigma = (R_s{\cdot}t)^{-1}$$

The conductivity of the material was calculated according to the above equation.

The electromagnetic interference shielding effectiveness (EMI SE), is a measure of blocking electromagnetic waves (EMW).

EMI SE is experimentally defined as the logarithmic ratio of incoming power (P_I) to transmitted power (P_T) [2] that is measured in decibel (dB),

$$\text{SE (dB)} = \log_{10}(P_I/P_t)$$

When an EM radiation is incident on shielding film, the incident power (P_I) can be expressed the added combination of the reflected power (P_R), absorbed power (P_A), and transmitted power (P_T)

$$P_I = P_R + P_A + P_T$$

For the intensity (I) it is,

$$I_O = I_R + I_A + I_T$$

Specific Shielding Effectiveness (SSE- dB $cm^3{\cdot}g^{-1}$) is mathematically expressed. Here, SSE is calculated dividing the EMI SE by the density of the material (ϱ).

$$SSE = \frac{EMI\ SE}{density}$$

SSE gives a more accurate account on EMI SE compare to ϱ of the material where thinner material might be having higher EMI SE [1–7].

The relationship between SE and thickness can be expressed in the following equation known as absolute effectiveness. Because SSE does not show thickness-based information while absolute effectiveness (SSE/t- dB cm^2·g^{-1}) is used to evaluate the relationship between SSE and thickness.

$$SSE/t = \frac{SSE}{t}$$

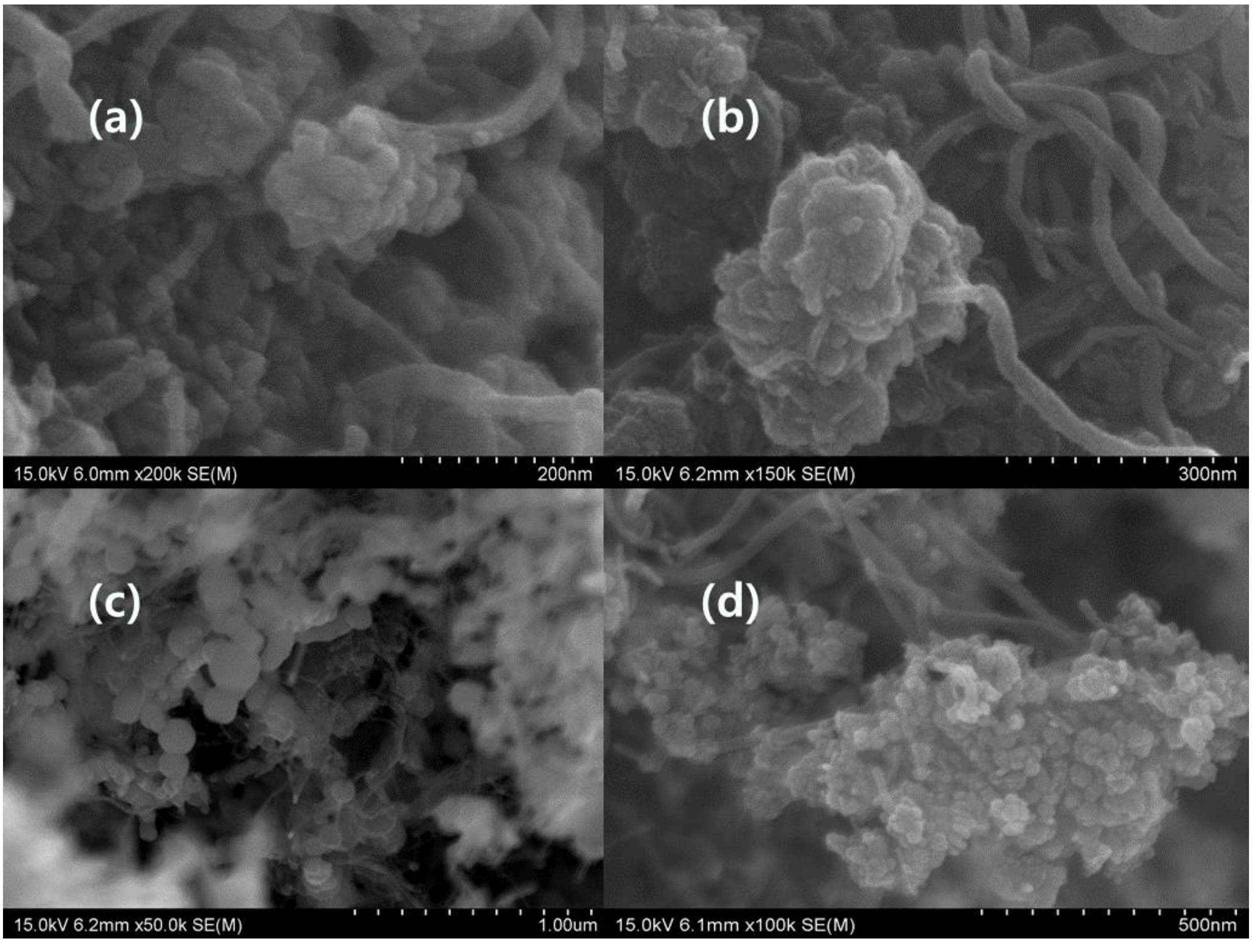

Figure S1. SEM image of CNTO decorated by (a) Fe_3O_4 (b) Fe (c) Ni (d) Cu.

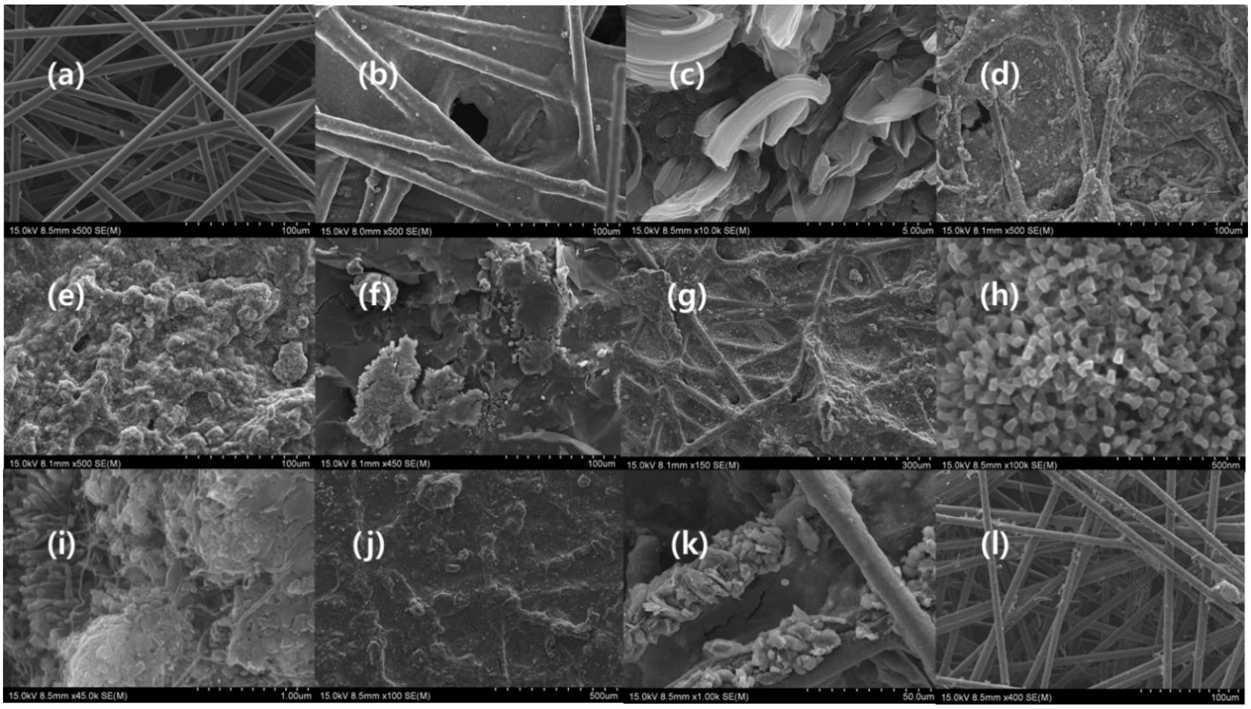

Figure S2. SEM image of carbon fabric composite of (a) MC (500) (b) MXCB (500) (c) surface of MXCS (10000) (d) MXCBCFeO (500) (e) MXCBCFe (500) (f) MXCBCNi (450) (g) MXCBCCo (150) (h) MXCBCCo (100000) (i) MXXBCCu (45000) (j) MXCNTC30 (100) (k) MXCNTNi25 (1000) MXCNT10 (400).

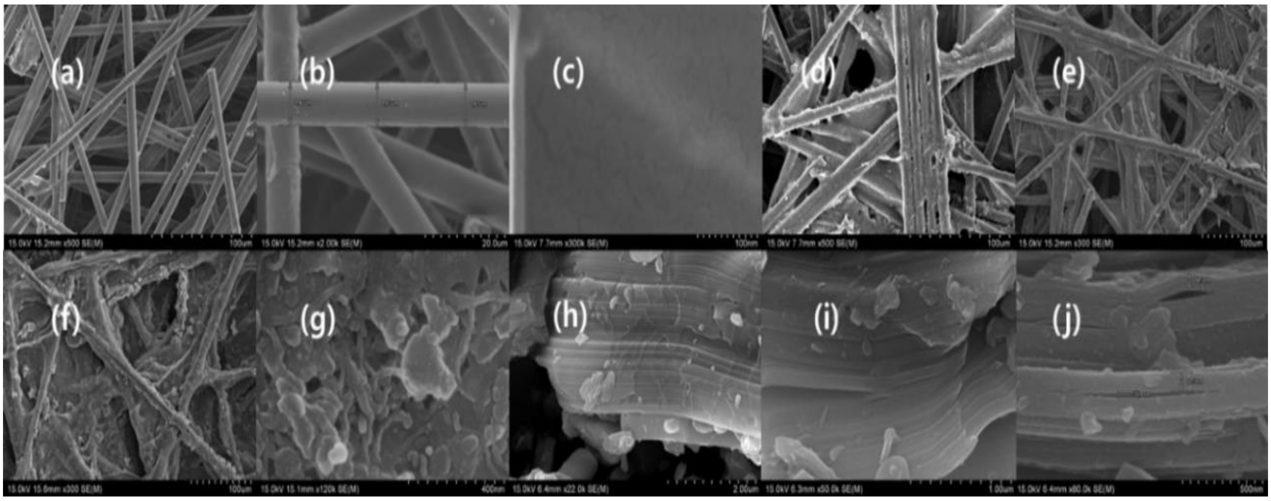

Figure S3. SEM image of (a) MC (×500), (b) MC (×2000), (c) cracks on fiber (×300000), (d) MXene-CNTO coated carbon fabric (×500), (e) MXene-CNTO coated carbon fabric (×300), (f) MXene-CNTO coated fabric (Ni coated fabric) (×300), (g) MXene and CNTO on the surface of the fabric (×120000), (h) Ti_3AlC_2 (×22000), (i) $Ti_3C_2T_x$ (×50000) and (j) $Ti_3C_2T_x$ (×80000).

Table S1. Elemental percentage of composites from EDX analysis.

**Elements**	**Ti%**	**Al%**	**C%**	**O%**	**F%**	**S%**	**Cl %**	**Br %**	**Co %**	**Ni %**	**Cu %**	**Fe %**
**MXCB**	0.53	2.7	71.13	5.36	20.26	-	-	-	-	-	-	-
**MXCBCFeO**	0.34	-	64.21	9.86	18.96	-	-	-	-	-	-	6.63
**MXCBCFe**	0.33	4.44	75.07	17.55	-	-	-	-	-	-	-	5.60
**MXCBNi**	-	-	86.66	9.01	3.68	-	-	-	-	0.65	-	-
**MXCBCCo**	0.38	-	64.82	15.96	15.05	-	-	-	3.78	-	-	-
**MXCBCCu**	0.04	-	94.34	-	-	-	-	4.10	-	-	1.52	-
**MXCS**	0.73	2.18	49.74	13.31	34.04	-	-	-	-	-	-	-
**FC**	-	-	89.24	10.76	-	-	-	-	-	-	-	-
**MC**	-	-	88.24	11.76	-	-	-	-	-	-	-	-
**MXCNTC30**	0.80	0.71	89.20	7.87	-	1.43	-	-	-	-	-	-
**MXCNTC25**	1.82	2.78	47.94	11.55	35.16	0.79	-	-	-	-	-	
**MXCNTNiC25**	1.04	2.45	53.52	9.66	30.21	0.74	-	-	-	2.39	-	-
**MXCNTC10**	0.39	0.47	79.38	7.87	11.89		-	-	-	-	-	-
**$Ti_3C_2T_x$**	16.36	1.74	12.81	9.42	59.09	-	0.59	-	-	-	-	-
**Ti_3AlC_2**	35.29	14.44	50.27	-	-	-	-	-	-	-	-	-

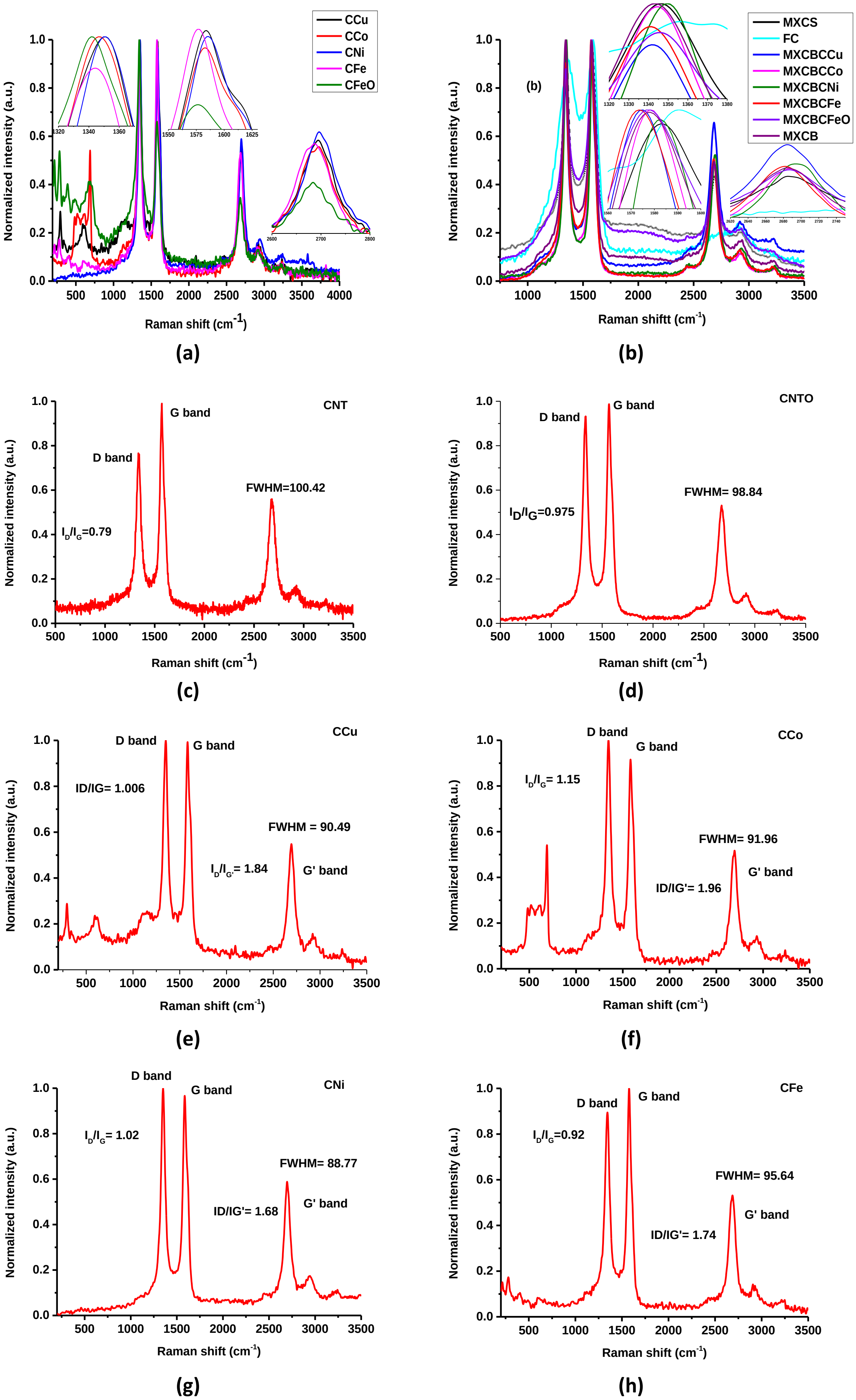

CCu
CCo
CNi
CFe
CFeO
Normalized intensity (a.u.)
Raman shift (cm-1)
(a)
MXCS
FC
MXCBCCu
MXCBCCo
MXCBCNi
MXCBCFe
MXCBCFeO
MXCB
(b)
Raman shiftt (cm-1)
CNT
D band
G band
FWHM=100.42
ID/IG=0.79
(c)
CNTO
D band
G band
FWHM= 98.84
ID/IG=0.975
(d)
CCu
D band
G band
ID/IG= 1.006
FWHM = 90.49
ID/IG'= 1.84
G' band
(e)
CCo
D band
G band
ID/IG= 1.15
FWHM= 91.96
ID/IG'= 1.96
G' band
(f)
CNi
D band
G band
ID/IG= 1.02
FWHM= 88.77
ID/IG'= 1.68
G' band
(g)
CFe
D band
G band
ID/IG=0.92
FWHM= 95.64
ID/IG'= 1.74
G' band
(h)

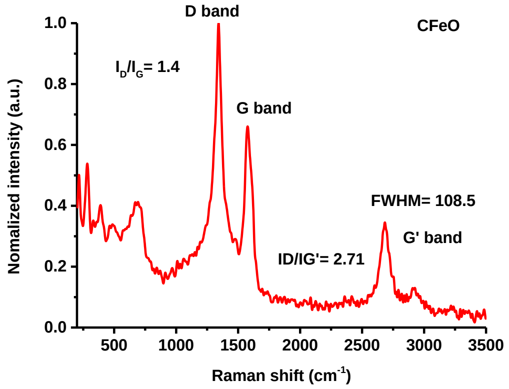
CFeO
D band
G band
G' band
I_D/I_G= 1.4
FWHM= 108.5
ID/IG'= 2.71
Nomalized intensity (a.u.)
Raman shift (cm^{-1})

(i)

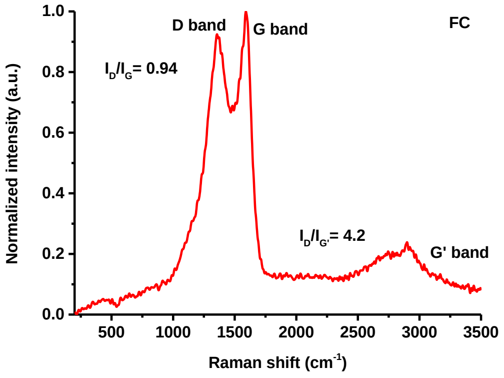
FC
D band
G band
G' band
I_D/I_G= 0.94
$I_D/I_{G'}$= 4.2
Normalized intensity (a.u.)
Raman shift (cm^{-1})

(j)

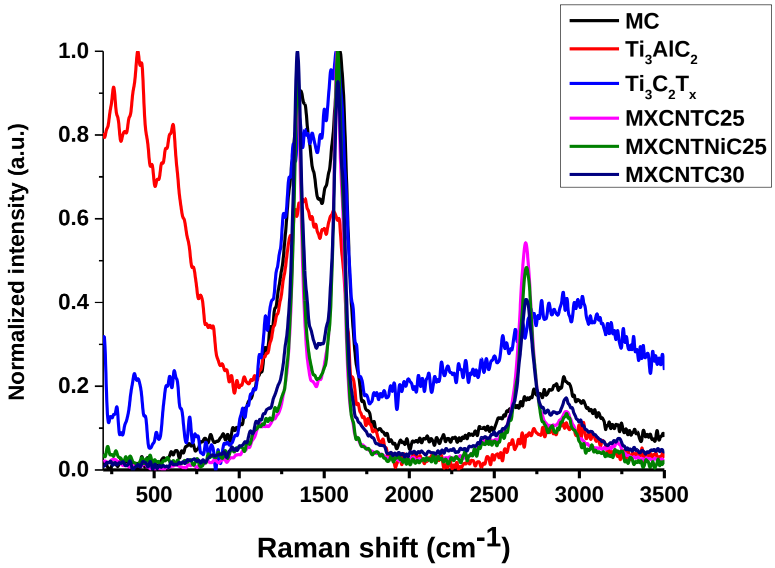
MC
Ti_3AlC_2
$Ti_3C_2T_x$
MXCNTC25
MXCNTNiC25
MXCNTC30
Normalized intensity (a.u.)
Raman shift (cm^{-1})

(k)

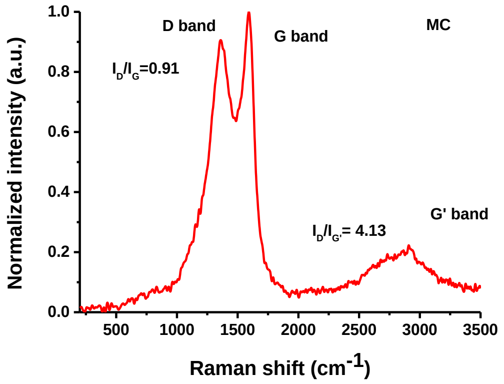
MC
D band
G band
G' band
I_D/I_G=0.91
$I_D/I_{G'}$= 4.13
Normalized intensity (a.u.)
Raman shift (cm^{-1})

(l)

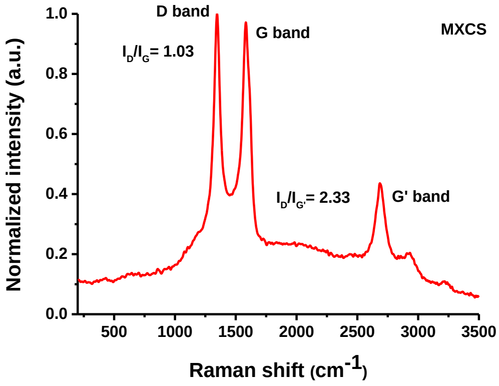
MXCS
D band
G band
G' band
I_D/I_G= 1.03
$I_D/I_{G'}$= 2.33
Normalized intensity (a.u.)
Raman shift (cm^{-1})

(m)

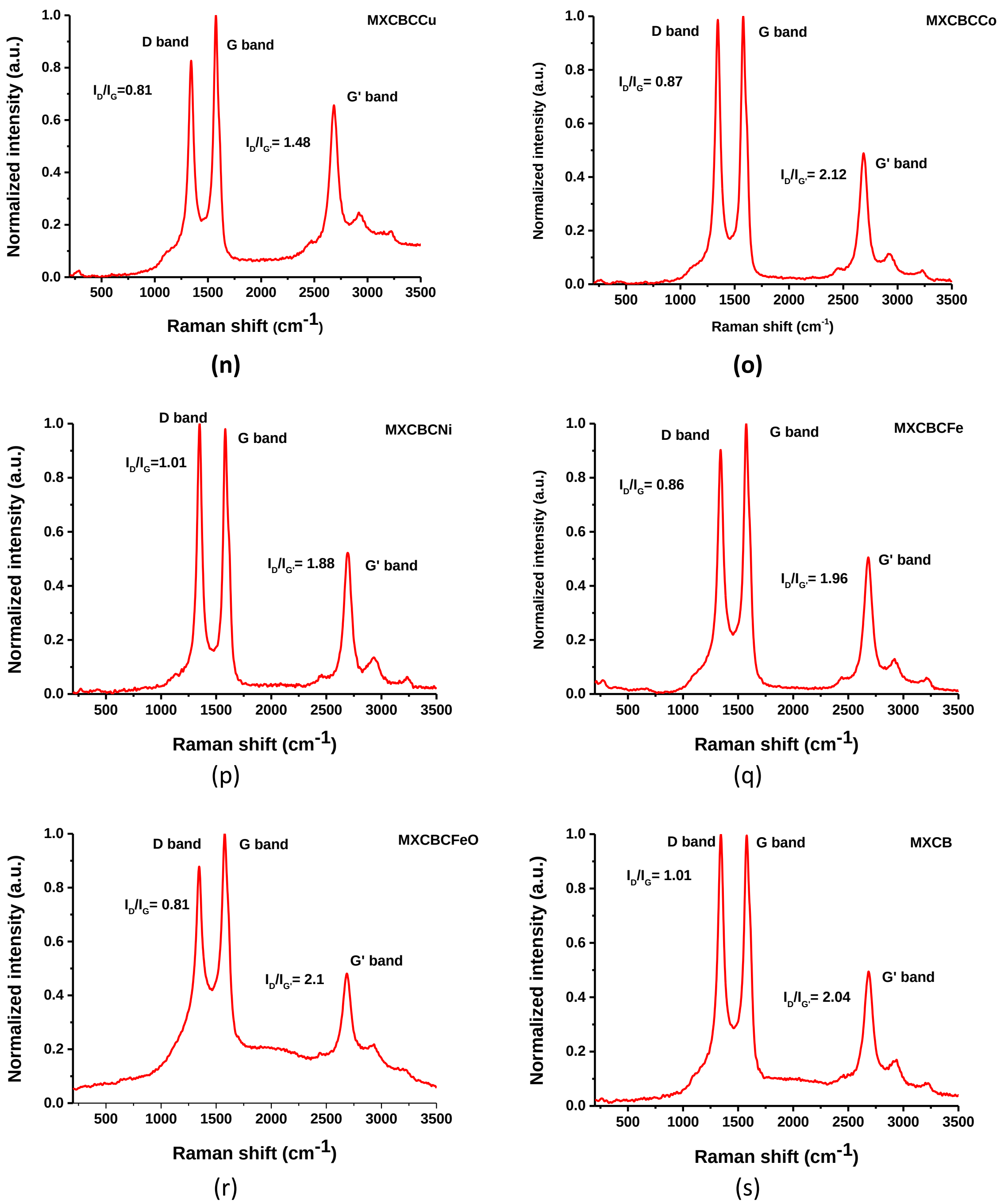

Figure S4. Normalized curve of Raman spectrum of (a) decorated CNT, (b) composites (c) CNT, (d) CNTO, (e) CCu, (f) CCo, (g) CNi, (h) CFe, (i) CFeO, (j) FC (k) comparison of MXene, MAX phase and MC, (l) MC, (m) MXCS, (n) MXCBCCu, (o) MXCBCCo, (p) MXCBCNi, (q) MXCBCFe, (r) MXCBCFeO and (s) MXCB.

Table S2. Raman spectra band positions.

NOs	Compositions	D band (cm^{-1})	G band (cm^{-1})	G′ band (cm^{-1})	ID/IG	ID/IG′	
1	CNT	1336.3	1570.9	2675.9	0.79	1.39	2933, 3220.7
2	CNTO	1341.5	1570.9	2675.9	0.96	1.76	2916.7, 3228.8
3	CCu	1354.6	1583.5	2693.4	1.01	1.84	297, 609, 843.5, 1138, 2922.4, 3239.9
4	CCo	1346.8	1577.8	2699.1	1.15	1.96	482, 520.3, 609.7, 693.3, 2936.5, 3237.8
5	CNi	1344	1584.2	2699.1	1.02	1.68	2942.9, 3237.75
6	CFe	1346.8	1577.8	2686.3	0.92	1.74	212.8, 276.5, 404.8, 603.3, 2911, 2232
7	CFeO	1340.4	1577.8	2680	1.4	2.71	219.1, 282.9, 382.9, 334.7, 482.1, 1475.1, 2917.4, 3225
8	FC	1354.57	1589.9	2899	0.94	4.18	-
9	MC	1363.1	1592	2908.2	0.91	4.13	
10	MXCS	1348.9	1583.5	2687.8	1.03	2.33	2922.4, 3210.12
11	MXCBCCu	1343.23	1577.83	2687.8	0.81	1.48	285.8, 2470.2, 2916.7, 3227.8
12	MXCBCo	1348.2	1576.4	2687.8	0.87	2.12	255.3, 428.2, 2451.03, 2925.2, 3225.7
13	MXCBNi	1348.2	1585.6	2697	1.01	1.88	263.8, 419, 582.7, 665, 2451, 2925.2, 3235
14	MXCBCFe	1339.7	1567.2	2678.5	0.86	1.96	273, 655.7, 2441.8, 2916, 3207.3
15	MXCBCFeO	1339.7	1576.4	2687.8	0.81	2.1	2441.8, 2933.7, 3207.3
16	MXCB	1348.2	1576.42	2687.8	1.01	2.04	263.8, 2441.8, 2933.7, 3216.5

Table S3. Atomic percentage MXene, MC, CNT, CNTO and composites from XPS analysis.

**Elements**	**C1s %**	**O1s %**	**F1s %**	**Ti %**	**N**	**S**	**Si**	**Cl**	**Fe**	**Ni**	**Co**	**Cu**
**MC**	89.54	8.8	-	-	1.16		0.51	-				
**CNT**	98.48	1.52	-	-	-		-					
**CNTO**	98.27	1.73	-	-	-		-					
**MXCNTC25**	82.98	11.29	4.19	1.53	-		-					
**MXene**	20.49	12.81	61.65	4.27	-		-	0.77				
**MXCB**	79.88	8.91	6.56	2.52	2.13							
**MXCBCFeO**	84.19	8.28	2.56	0.29	2.16		-	-	2.03			
**MXCBCFe**	74.12	17.22	2.48	0.71	2.31		-	-	3.15			
**MXCBCNi**	66.8	16.95	8.44	1.07	2.16		-	-	-	4.58		
**MXCBCCo**	80.52	7.25	7.68	1.09	2.15		-	-	-	-	1.3	
**MXCBCCu**	77.47	8.7	8.76	1.71	2.01							1.35
**MXCNTC25**	78.19	13.92	3.54	1.18	-	2.4						
**MXCNTNiC25**	77.9	-	12.55	0.69		2.78						
**FC**	84.72	11.65			2.87							
**MXCS**	65.91	18.93	4.43	1.35	0.97							

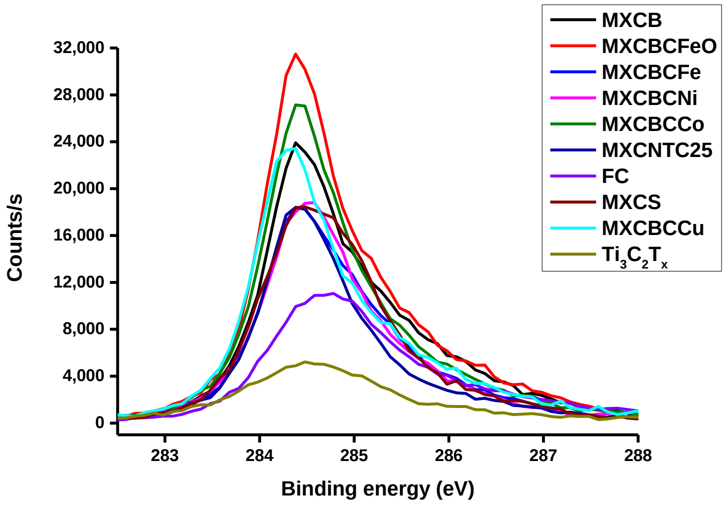

Figure S5. The c1s fitting curve of the MXene, functionalized fabric and composites.

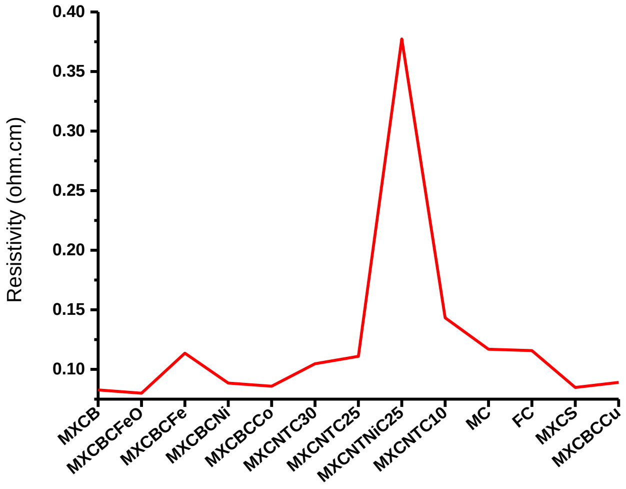

Figure S6. Resistivity profile of the composites.

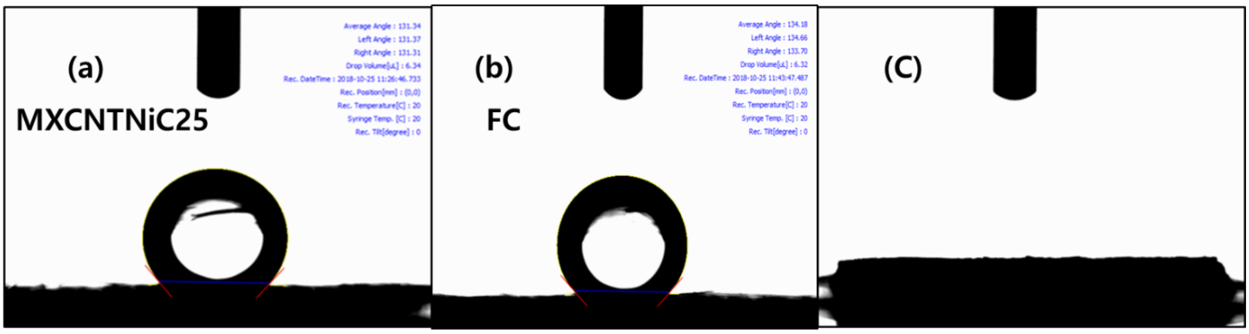

Figure S7. Contact angle of (a) MXCNTNiC25 (b) FC and (c) represent other composites.

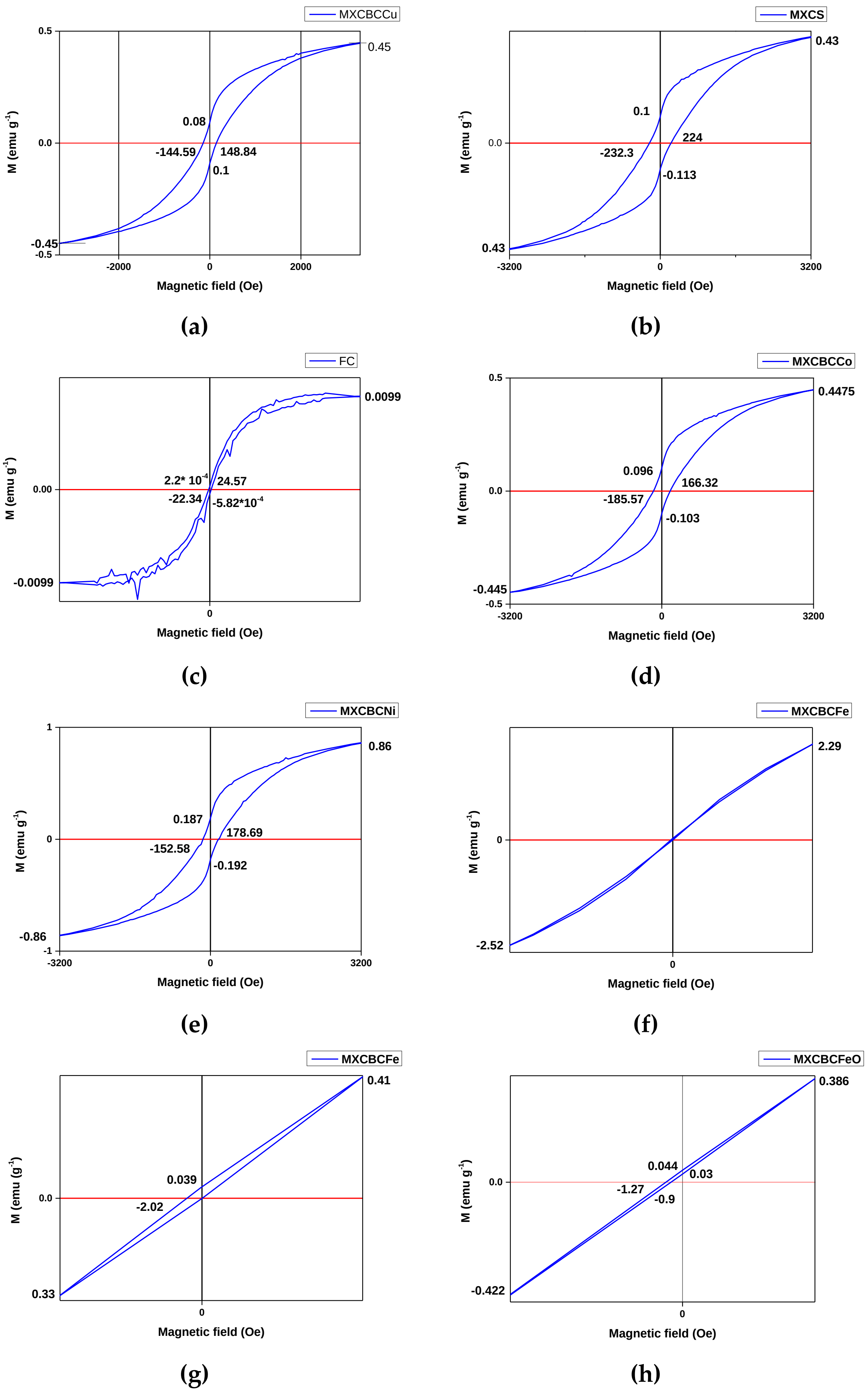

Figure S8. magnetization of composites against the applied field at 300 K (a) MXCBCCu, (b) MXCS, (c) FC, (d) MXCBCCo, (e) MXCBCNi, (f) MXCBCFe, (g) MXCBCFe and (h) MXCBCFeO.

Table S4. Comparison of EMI SE with thickness.

	Compositon	Filler	t(mm)	SE (dB)	SSE(dB cm^3g^{-1})	SSE/t ($dBcm^2 g^{-1}$)	Ref.
1	GN/Fe_3O_4	0	0.3	24	31	1033	7
2	MWCNT	PC	2.1	39	34.5	154	8
3	CB	EPDM	2	18	30.3	15.1	9
4	CB	ABS	1.1	20	20.9	190	10
5	MWCNT	PS	2	30	57	285	11
6	CNT	POLYM	3.5	80	*	*	12
7	Cu foil	0	0.01	70	7.8	7812	2
8	Al foil	0	0.008	66	24.4	30555	2
9	stainless steel	0	4	89	11	27.5	13
10	MWCNT/NCF	0	0.138	28.22	486.54	35256	14
11	NCF-20 g/m^2	0	0.127	25.56	381.50	30039	14
12	Cu bulk	0	3.1	90	10	32.3	13
13	Ni fiber	PES	2.85	58	31	108.7	13
14	Mxene	PET	0.045	92	138.735	30,830	2
15	MXene	cellusolse	0.047	25.8	12.44	2647	15
16	Mxene/foam		0.06	70	*	*	16
17	Mxene	parafine	1	76.1	*	*	17
18	Graphene	PVDF/MWCNTs (PF/CNT)	2	28.5	*	*	18
19	rGO	$PbTiO_3$/PEDOT	2.5	51.94	*	*	19
20	CNT	polypropylene	2.2	48.3	*	*	20
21	Graphene nanoplate	B_4C	2	38	*	*	21
22	Graphene	PVDF	3	37.4	*	*	22
23	Large size graphene (LG)	Doping by iodine	0.0125	52.2	*	*	23
24	Expanded graphite (EG)	Large Flexible Graphene	0.043	48.3	*	*	24
25	GNP	PBAT	1	14	*	*	25
26	Porous Fe_3O_4	C	4.27	54.6	*	*	26
27	CF-30 g/m^2	0	0.219	27.14	381.5	30039	27
28	MGNC-S band	polymer	0.35	43.2	46.4	1324.29	
29	MGNC-X band	polymer	0.35	53.88			27
30	Polystyrene (PS)	Carbonaceous Filler	27	22	92	*	28

31	Poly propylene (PP)	MWCNT-S band	2	57	*	*	29
32	PP	MWCNT-Ku band	2	44	*	*	29
33	PP	MWCNT-X band	2	47	*	*	29
34	PP	Graphene- S band	0.5	13	*	*	30
35	PP/PS	SBS	2	24.9	*	*	31
36	rGNO	SiO_2	1.5	37	*	*	32
37	CNF/CNT	PS	1	21.9	*	*	33
38	MXene (Ti3C2TX)	PS-570	2	62	*	*	34
39	MXene	PS-570	1.5	44	*	*	34
40	MXene	PS-570	1	27	*	*	34
41	MXSC	CNTO		50.5			This work
* Sign indicates that the values were impossible to calculate or not available enough data to calculate. Densities of MC and FC were 0.146 g cm^{-3} and 0.108 g cm^{-3} respectively							

Table S5. Comparison of maximum (MAX), minimum (MINI), average (AVE) shielding, SSE and SSE/t of the composite in each case.

Band	Types of shielding		MXCB	MXCB CFeO	MXCB CFe	MXCB CNi	MXCB CCo	MXB CCu	MXCS
X-band	SE	Max	47.6	45.9	46.7	45	46	43.6	50.5
		Min	46.8	45.2	45.7	44.2	45.2	42.8	49.2
		Ave	47.1	45.4	46.1	44.4	45.4	43	49.6
	SER	Max	14.6	13.7	12.7	14.3	14.3	12.9	13.7
		Min	12.8	11.4	10.5	12.3	12.4	10.6	11.8
		Ave	13.5	12.3	11.3	13.1	13.1	11.5	12.5
	SEA	Max	34.7	34.3	36.3	32.4	33.4	32.7	38.7
		Min	32.9	32.2	33.5	30.7	31.7	30.7	35.8
		Ave	33.6	33.1	34.7	31.3	32.3	31.5	37.1
			MXCNT C30	MXCNT C25	MXCNTC NiC25	MXCNT C10	MC	FC	-
X-band	SE	Max	47.3	47.1	34.9	39.9	31.7	43.9	-
		Min	46.5	46.2	32.9	39.3	29.45	43.1	-
		Ave	46.7	46.5	33.5	39.4	30.15	43.4	-
	SER	Max	14.5	12.7	13.1	11.2	11.73	14	-
		Min	12.4	10.5	11.3	9.3	10.31	12.1	-
		Ave	13.2	11.4	12	10	10.78	12.8	-
	SEA	Max	35	36.6	21.9	30.6	20.01	31.8	-
		Min	32.5	34	21.4	28.5	19.1	29.8	-
		Ave	33.6	35.1	21.5	29.4	19.37	30.6	-
			MXCNT C30	MXCNT C25	MXCNT NiC25	MXCNT C10	MC		-
S-band	SE	Max	39.6	39.9	34.1	33.2	28.5		-
		Min	35.3	32.5	20.9	30.1	23.2		-
		Ave	36.8	35.7	28.4	31.2	25.6		-

Table S6. Density of the composites.

Composites	Density (g cm^{-3})	Thickness (mm)	Surface Resistance (Ω/sq)	Resistivity (Ω cm)	Conductivity (S/cm)	SSE (dBcm^{3}g^{-1})	SSE/t (dBcm^{2}g^{-1})
MXCB	0.229	0.398	2.08	0.08269	12.1	205.52	5163.75
MXCBCFeO	0.288	0.362	2.21	0.08003	12.5	157.75	4357.67
MXCBCFe	0.269	0.38	2.99	0.11356	8.81	171.13	4503.53
MXCBCNi	0.205	0.372	2.38	0.08854	11.3	216.55	5821.15
MXCBCCo	0.257	0.408	2.11	0.08587	11.65	176.7	4330.82
MXCNTC30	0.212	0.378	2.77	0.10473	9.55	220.5	5833.34
MXCNTC25	0.167	0.398	2.78	0.11092	9.02	278.35	6993.78
MXCNTNiC25	0.224	0.27	13.98	0.37737	2.65	149.37	5532.29
MXCNTC10	0.150	0.288	4.98	0.14332	6.98	262.4	9110.97
MC	0.146	0.266	4.4	0.11692	8.55	206.48	7762.5
FC	0.108	0.318	3.64	0.11574	8.64	401.93	12639.35
MXCS	0.153	0.386	2.2	0.08478	11.8	324.15	8397.78
MXCBCCu	0.205	0.348	2.56	0.0891	11.22	209.66	6024.84

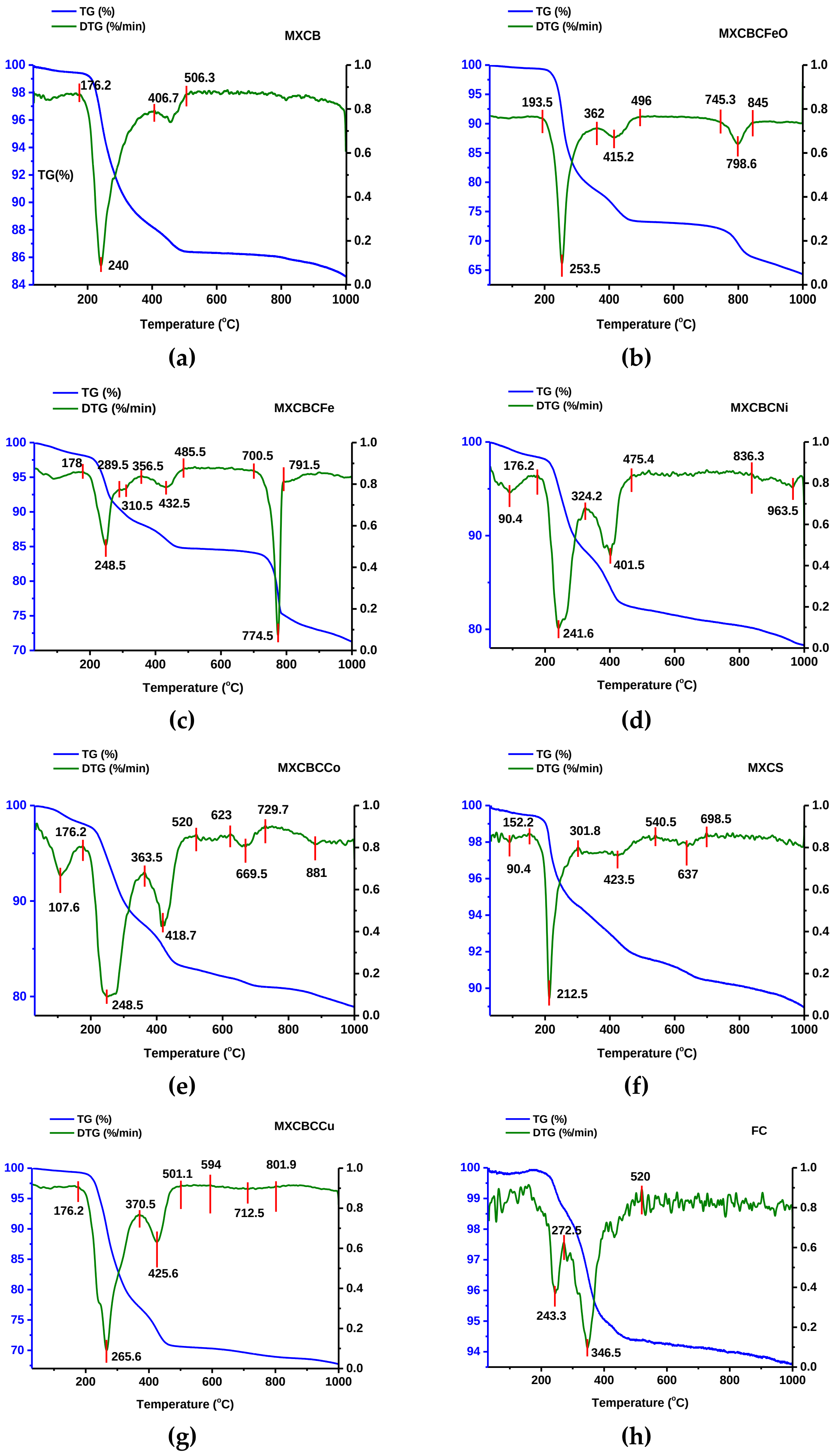

TG (%)
DTG (%/min)
MXCB
TG(%)
176.2
240
406.7
506.3
Temperature (°C)
(a)
TG (%)
DTG (%/min)
MXCBCFeO
193.5
253.5
362
415.2
496
745.3
798.6
845
Temperature (°C)
(b)
TG (%)
DTG (%/min)
MXCBCFe
178
248.5
289.5
310.5
356.5
432.5
485.5
700.5
774.5
791.5
Temperature (°C)
(c)
TG (%)
DTG (%/min)
MXCBCNi
176.2
90.4
241.6
324.2
401.5
475.4
836.3
963.5
Temperature (°C)
(d)
TG (%)
DTG (%/min)
MXCBCCo
176.2
107.6
248.5
363.5
418.7
520
623
669.5
729.7
881
Temperature (°C)
(e)
TG (%)
DTG (%/min)
MXCS
152.2
90.4
212.5
301.8
423.5
540.5
637
698.5
Temperature (°C)
(f)
TG (%)
DTG (%/min)
MXCBCCu
176.2
265.6
370.5
425.6
501.1
594
712.5
801.9
Temperature (°C)
(g)
TG (%)
DTG (%/min)
FC
243.3
272.5
346.5
520
Temperature (°C)
(h)

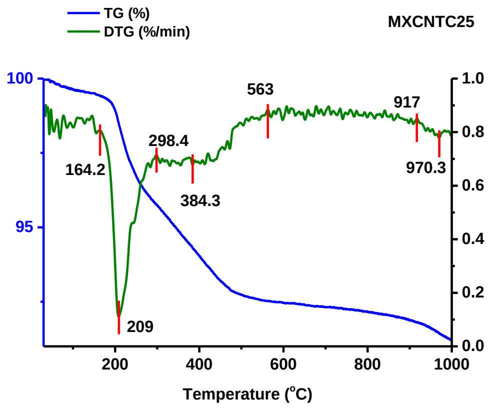

(i)

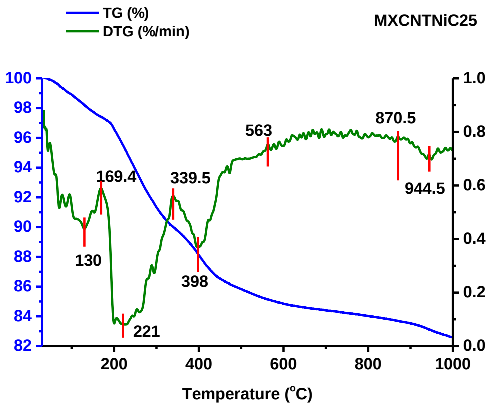

(j)

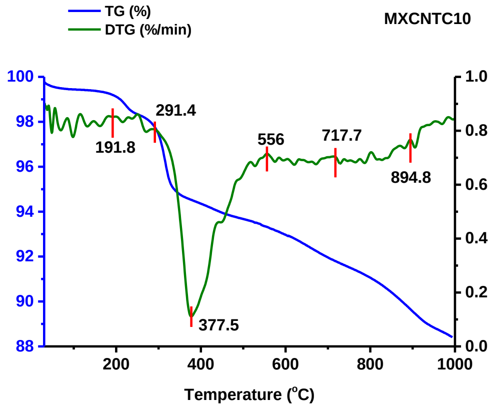

(k)

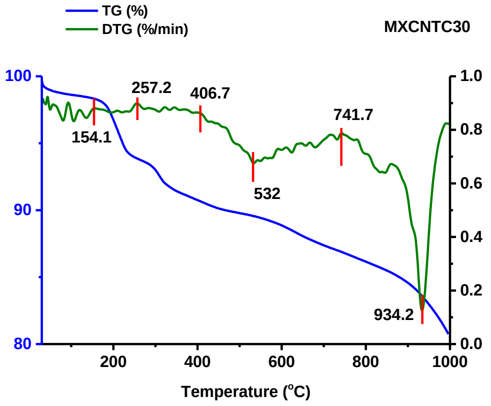

(l)

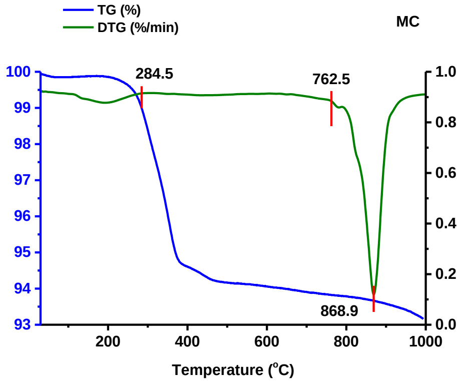

(m)

Figure S9. Comparison of the TG and DTA of all the composites (a) MXCB, (b) MXCBCFeO, (c) MXCBCFe, (d) MXCBCNi, (e) MXCBCCo, (f) MXCS, (g) MXCBCCu, (h) FC, (i) MXCNTC25, (j) MXCNTNiC25, (k) MXCNTC10, (l) MXCNTC30 and (m) MC.

Table S7. Comparison of mass changes with different temperature range from TGA analysis.

Nos	Compositions	Temperature range (℃)	Mass change (%)	Beginning of Degradation temperature (℃)	Rapid mass change temperature (℃)	Total mass change (%)
1	MXCB	30-162	0.56			
		162-412.5	11.28	162	162-580	15.42
		412.5-580	1.84			
		580-1000	1.74			
2	MXCBCFeO	30-152	0.63			
		152-361	20.84	152	152-576	35.71
		361-576.5	5.52			
		576.5-1000	8.72			
3	MXCBCFe	30-160.5	1.88			
		160.5-348.5	9.88	160.5	160.5-652.5	28.76
		348.5-652.5	3.69			
		652.5-847.5	9.91			
		847.5-1000	3.40			
4	MXCBCNi	30-185	1.60			
		185-326	10.03	185	185-530	21.71
		326-530	6.34			
		530-780	1.64			
		780-1000	2.10			
5	MXCBCCo	30-181	1.91			
		181-345	10.60	181	181-549	21.8
		345-549	4.94			
		549-796	1.63			
		796-1000	2.00			
6	MXCS	30-148.5	0.57			
		148.5-293.5	4.92	148.5	148.5-514	11.08
		293.5-514	2.97			
		514-778.5	1.31			
		778.5-1000	1.31			
7	MXCBCCu	30-127.5	0.63			
		127.5-367	22.20	127.5	127.5-564	32.27
		367-564	6.78			
		564-885.4	1.75			
		885.4-1000	0.91			
8	MXCNTC10	30-146.5	0.57			
		146.5-260.5	1.12			
		260.5-390	3.81	146.5	146.5-761	11.61
		390-500.5	0.82			
		500.5-761	2.12			
		761-1000	3.19			
9	MXCNTC25	30-145.5	0.58			
		145.5-283	3.64	145.5	145.5-701	8.85
		283-701	3.44			
		701-1000	1.19			
10	MXCNTNiC25	30-162	2.58			
		162-336.5	7.40	162	162-897.5	17.47

		336.5-897.5	5.74			
		897.5-1000	1.75			
11	MXCNTC30	30-120	1.42			
		120-247	4.85	120	120-771.5	19.3
		247-506	3.89			
		506-771.5	2.78			
		771.5-1000	6.3			
12	MC	30-190	0.11			
		190-395	5.29	190	190-395	6.84
		395-1000	1.44			
13	FC	30-255.5	1.34	255.5	255.5-588	6.4
		255.5-588.5	4.39			
		588.5-1000	0.67			

Table S8. Comparison of different peak positions range from DTA analysis.

NOs	Composites	Different peak range (℃)	The lowest point of the prominent peak (℃)
1	MXCB	30-176.2,	
		176.2-406.7	240
		406.7-506.3	
		506.3-1000	
2	MXCBCFeO	30-193.5	
		193.5-362	253.5
		362-496	415.2
		745.3-845	798.6
3	MXCBCFe	30-178	
		178-289.5	248.5
		289.5-356.5	310.5
		356.5-485.5	432.5
		700.5-791.5	774.5
4	MXCBCNi	30-176.2	90.4
		176.2-324.2	241.6
		324.2-475.4	401.5
		475.4-836.3	
		836.3-1000	963.5
5	MXCBCCo	30-176.2	107.6
		176.2-363.5	248.5
		363.5-520	418.7
		623-729.7	669.5
		729.7-1000	881
6	MXCS	30-152.2	90.4
		152.2-301.8	212.5
		301.8-540.5	423.5
		540.5-698.5	637
		698.5-1000	
7	MXCBCCu	30-176.2	
		176.2-370.5	265.6
		370.5-501.1	425.6
		594-801.9	712.5

		801.9-1000	
8	MXCNTC10	30-191.8	
		191.8-291.4	
		291.4-556	377.5
		556-717.7	
		717.7-894.8	
		894.8-1000	
9	MXCNTC25	30-164.2	
		164.2-298.4	209
		298.4-563	384.3
		917-1000	970.3
10	MXCNTNiC25	30-169.4	130
		169.4-339.5	221
		339.5-563	398
		870.5-1000	944.5
11	MXCNTC30	30-154.1	
		154.1-257.2	
		257.2-406.7	
		406.7-741.7	532
		741.7-1000	934.2
12	MC	30-284.5	
		284.5-762.5	
		762.5-1000	868.9
13	FC	30-272.5	243.3
		272.5-520	272.5

References

1. Ameli, A.; Nofar, M.; Wang, S.; Park, C.B. Lightweight Polypropylene/Stainless-Steel Fiber Composite Foams with Low Percolation for Efficient Electromagnetic Interference Shielding. *Appl. Mater. Interfaces* **2014**, *6*, 11091–11100.
2. Shahzad, F.; Alhabeb, M.; Hatter, C.B.; Anasori, B.; Hong, S.M.; Koo, C.M.; Gogotsi, Y. Electromagnetic interference shielding with 2D transition metal carbides (MXenes). *Science* **2016**, *353*, 1137–1140.
3. Bian, X.M.; Liu, L.; Li, H.B.; Wang, C.Y.; Xie, Q.; Zhao, Q.L.; Bi, S.; Hou, Z.L. Construction of three-dimensional graphene interfaces into carbon fiber textiles for increasing deposition of nickel nanoparticles: flexible hierarchical magnetic textile composites for strong electromagnetic shielding. *Nanotechnology* **2017**, *28*, 45710.
4. Yan, D.X.; Pang, H.; Li, B.; Vajtai, R.; Xu, L.; Ren, P.G.; Wang, J.H.; Li, Z.M. Structured Reduced Graphene Oxide/Polymer Composites for Ultra-Efficient Electromagnetic Interference Shielding. *Adv. Funct. Mater.* **2015**, *25*, 559–566.
5. Zeng, Z.; Jin, H.; Chen, M.; Li, W.; Zhou, L.; Zhang, Z. Lightweight and Anisotropic Porous MWCNT/WPU Composites for Ultrahigh Performance Electromagnetic Interference Shielding *Adv. Funct. Mater*. **2016**, *26*, 303–310.
6. Z. Han, Z.; Fina, A. Thermal conductivity of carbon nanotubes and their polymer nanocomposites: A review. *Prog. Polym. Sci*. **2011**, *36*, 914–944.
7. Agnihotri, N.; Chakrabarti, K.; De, A.; Highly efficient electromagnetic interference shielding using graphite nanoplatelet/poly(3,4-ethylenedioxythiophene)-poly(styrenesulfonate) composites with enhanced thermal conductivity. *RSC Adv*. **2015**, *5*, 43765–43771.
8. Pande, S.; Chaudhary, A.; Patel, D.; Singh, B.P.; Mathur, R.B. Mechanical and electrical properties of multiwall carbon nanotube/polycarbonate composites for electrostatic discharge and electromagnetic interference shielding applications. *RSC Adv*. **2014**, *4*, 13839

9. Ghosh, P.; Chakrabarti, A. Conducting carbon black filled EVA vulcanizates: Assessment of dependence of physical and mechanical properties and conducting character on variation of filler loading. *J. Polym. Mater.* **2000**, *17*, 291–304.
10. Al-Saleh, M.H.; Saadeh, W.H.; Sundararaj, U. EMI shielding effectiveness of carbon based nanostructured polymeric materials: A comparative study. *Carbon N. Y.* **2013**, *60*, 146–156.
11. Arjmand, M.; Apperley, T.; Okoniewski, M.; Sundararaj, U. Comparative study of electromagnetic interference shielding properties of injection molded versus compression molded multi-walled carbon nanotube/polystyrene composites. *Carbon N. Y.* **2012**, *50*, 5126–5134.
12. Micheli, D.; Vricella, A.; Pastore, R.; Delfini, A.; Giusti, A.; Albano, M.; Primiani, V. M. Ballistic and electromagnetic shielding behaviour of multifunctional Kevlar fiber reinforced epoxy composites modified by carbon nanotubes. *Carbon* **2016**, *104*, 141-156.
13. Shui, X.; Chung, D.D.L. Nickel filament polymer-matrix composites with low surface impedance and high electromagnetic interference shielding effectiveness. *J. Electron. Mater*. **1997**, *26*, 928–934.
14. Pothupitiya Gamage, S.J., Yang, K., Braveenth, R., Raagulan, K., Kim, H.S., Lee, Y.S., Yang, C.M., Moon, J.J. and Chai, K.Y. MWCNT coated free-standing carbon fiber fabric for enhanced performance in EMI shielding with a higher absolute EMI SE. *Materials* **2017**, *10*, 1350.
15. Cao, W.T.; Chen, F.F.; Zhu, Y.J.; Zhang, Y.G.; Jiang, Y.Y.; Ma, M.G.; Chen, F. Binary Strengthening and Toughening of MXene/Cellulose Nanofiber Composite Paper with Nacre-Inspired Structure and Superior Electromagnetic Interference Shielding Properties. *ACS nano* **2018**, *12*, 4583–4593.
16. Liu, J.; Zhang, H.B.; Sun, R.; Liu, Y.; Liu, Z.; Zhou, A.; Yu, Z.Z. Hydrophobic, Flexible, and Lightweight MXene Foams for High-Performance Electromagnetic-Interference Shielding. *Adv. Mater.* **2017**, *29*, 1702367.
17. Han, M.; Yin, X.; Wu, H.; Hou, Z.; Song, C.; Li, X.; Zhang, L.; Cheng, L. Ti_3C_2 MXenes with modified surface for high-performance electromagnetic absorption and shielding in the X-band. *ACS Appl. Mater. Interfaces* **2016**, *8*, 21011–21019.
18. Ma, X.; Shen, B.; Zhang, L.; Liu, Y.; Zhai, W.; Zheng, W. Porous superhydrophobic polymer/carbon composites for lightweight and self-cleaning EMI shielding application. *Compos. Sci. Technol.* **2018**, *158*, 86–93.
19. Dalal, J.; Lather, S.; Gupta, A.; Dahiya, S.; Maan, A.S.; Singh, K.; Dhawan, S.K.; Ohlan, A. EMI shielding properties of laminated graphene and $PbTiO_3$ reinforced poly (3, 4-ethylenedioxythiophene) nanocomposites. *Compos. Sci. Technol.* **2018**, *165*, 222–230.
20. Wu, H.Y.; Jia, L.C.; Yan, D.X.; Gao, J.F.; Zhang, X.P.; Ren, P.G.; Li, Z.M. Simultaneously improved electromagnetic interference shielding and mechanical performance of segregated carbon nanotube/polypropylene composite via solid phase molding. *Compos. Sci. Technol.* **2018**, *156*, 87–94.
21. Tan, Y.; Luo, H.; Zhang, H.; Zhou, X.; Peng, S. Lightweight graphene nanoplatelet/boron carbide composite with high EMI shielding effectiveness. *AIP Adv.* **2016**, *6*, 035208.
22. Zhao, C.; Hamidinejad, M.; Wang, C.; Li, R.; Wang, S.; Yasamin, K.; Park, C.B. Incorporating a microcellular structure into PVDF/graphene-nanoplatelet composites to tune their electrical conductivity and electromagnetic interference shielding properties. *J. Mater. Chem. C* **2018**, *6*, 10292–10300.
23. Wan, Y.J.; Zhu, P.L.; Yu, S.H.; Sun, R.; Wong, C.P.; Liao, W.H., Graphene paper for exceptional EMI shielding performance using large-sized graphene oxide sheets and doping strategy. *Carbon* **2017**, *122*, 74–81.
24. Liu, Y.; Zeng, J.; Han, D.; Wu, K.; Yu, B.; Chai, S.; Chen, F.; Fu, Q.; Graphene enhanced flexible expanded graphite film with high electric, thermal conductivities and EMI shielding at low content. *Carbon* **2018**, *133*, 435–445.
25. Kashi, S.; Hadigheh, S.A.; Varley, R. Microwave Attenuation of Graphene Modified Thermoplastic Poly (Butylene adipate-co-terephthalate) Nanocomposites. *Polymers* **2018**, *10*, 582.
26. Wu, N.; Liu, C.; Xu, D.; Liu, J.; Liu, W.; Shao, Q.; Guo, Z. Enhanced electromagnetic wave absorption of three-dimensional porous Fe_3O_4/C composite flowers. *ACS Sustain. Chem. Eng. **2018***, 6, 12471–12480
27. Raagulan, K.; Braveenth, R.; Jang, H.; Seon Lee, Y.; Yang, C.M.; Mi Kim, B.; Moon, J.; Chai, K. Electromagnetic Shielding by MXene-Graphene-PVDF Composite with Hydrophobic, Lightweight and Flexible Graphene Coated Fabric. *Materials* **2018**, *11*, 1803.
28. Min, Z.; Yang, H.; Chen, F.; Kuang, T. Scale-up production of lightweight high-strength polystyrene/carbonaceous filler composite foams with high-performance electromagnetic interference shielding. *Mater. Lett*. **2018**, *230*, 157–160.
29. George, G.; Simon, S.M.; Prakashan, V.P.; Sajna, M.S.; Faisal, M.; Chandran, A.; Wilson, R.; Biju, P.R.; Joseph, C.; Unnikrishnan, N.V. Morphological, dielectric, tunable electromagnetic interference shielding and thermal characteristics of multiwalled carbon nanotube incorporated polymer nanocomposites: A facile, environmentally benign and cost effective approach realized via polymer latex/waterborne polymer as matrix. *Polym. Compos*. **2018**, *39*, E1169–E1183.

30. Huang, C.L.; Lou, C.W.; Liu, C.F.; Huang, C.H.; Song, X.M.; Lin, J.H. Polypropylene/graphene and polypropylene/carbon fiber conductive composites: Mechanical, crystallization and electromagnetic properties. *Appl. Sci.* **2015**, *5*, 1196–1210.
31. Al-Saleh, M.H.; Sundararaj, U. Electromagnetic interference (EMI) shielding effectiveness of PP/PS polymer blends containing high structure carbon black. *Macromol. Mater. Eng*. **2008**, *293*, 621–630.
32. Wen, B.; Cao, M.; Lu, M.; Cao, W.; Shi, H.; Liu, J.; Wang, X.; Jin, H.; Fang, X.; Wang, W.; Yuan, J. Reduced graphene oxides: light-weight and high-efficiency electromagnetic interference shielding at elevated temperatures. *Adv. Mater*. **2014**, *26*, 3484–3489.
33. Yang, Y.; Gupta, M.C.; Dudley, K.L. 2007. Towards cost-efficient EMI shielding materials using carbon nanostructure-based nanocomposites. *Nanotechnology* **2007**, *18*, 345701.
34. Sun, R.; Zhang, H.B.; Liu, J.; Xie, X.; Yang, R.; Li, Y.; Hong, S.; Yu, Z.Z. Highly conductive transition metal carbide/carbonitride (MXene)@ polystyrene nanocomposites fabricated by electrostatic assembly for highly efficient electromagnetic interference shielding. *Adv. Funct. Mater*. **2017**, *27*, 1702807.
